# Cannabinoid type 2 receptor regulates skeletal muscle regeneration by NLRP3-GSDMD mediated macrophage pyroptosis after injury

**DOI:** 10.1038/s41420-026-03077-z

**Published:** 2026-03-27

**Authors:** Xinjie Li, Haomiao Yuan, Shuyang Mu, Jiaqing Pan, Fuyuan Zhang, Shukui Du, Jin Liu, Anran Qu, Yingfu Sun, Linlin Wang, Ping Huang, Rui Zhao, Dawei Guan

**Affiliations:** 1 Department of Forensic Pathology, China Medical University School of Forensic Medicine, Shenyang, China; 2 Institute of Forensic Science, Fudan University, Shanghai, China; 3 Department of Emergency Medicine, Shengjing Hospital of China Medical University, Shenyang, China; 4 Liaoning Provincial Key Laboratory of Forensic Bio- evidence, Shenyang, China

**Keywords:** Regeneration, Cell death and immune response, Inflammasome

## Abstract

Skeletal muscle repair after injury requires coordinated immune responses. The cannabinoid type 2 receptor (CB2R) has been implicated in this process; however, its molecular mechanism in regulating inflammation and muscle regeneration, particularly whether it involves modulating macrophage pyroptosis—a specific pro-inflammatory cell death—remains elusive. This study proposes and validates a novel mechanism: CB2R activation protects skeletal muscle by inhibiting the PI3K/AKT/NF-κB signaling axis, thereby suppressing NLRP3 inflammasome-mediated pyroptosis in macrophages and ultimately fostering a pro-regenerative microenvironment. Using a mouse contusion model and conditioned medium assays, we demonstrate that CB2R deficiency exacerbates macrophage pyroptosis, elevates inflammatory mediators, and impairs muscle repair. This effect is driven by hyperactivation of the PI3K/AKT/NF-κB pathway, as blocking this pathway alleviated the inflammatory response and restored the expression of muscle regeneration markers. Furthermore, inflammatory signals released from CB2R-deficient macrophages directly impaired the development of muscle cells in the conditioned medium-based assay. Our findings uncover a novel non-cell-autonomous mechanism whereby CB2R supports skeletal muscle regeneration by restraining macrophage-driven inflammation and maintaining a repair-permissive environment, providing new insights into skeletal muscle repair from the perspective of the regenerative microenvironment and further expand the current understanding of muscle regeneration following injury. This work provides a robust mechanistic rationale for repurposing CB2R agonists as promising therapeutic strategies for muscle injury.

## Introduction

Skeletal muscle, especially those located near the body surface, is highly prone to mechanical injuries, with contusion being the most common type [1]. Muscle contusion typically results from blunt trauma, leading to muscle fiber rupture, hemorrhage, and sterile inflammation [2]. This sets off a complex regenerative process involving immune responses and tissue remodeling [3]. The gastrocnemius muscle is a standard injury model due to its accessibility and reproducible damage response. Successful muscle regeneration relies on the coordinated activation of satellite cells [4] and the dynamic response of macrophages [5]. Following injury, monocytes are recruited to the site and differentiate into pro-inflammatory macrophages that clear necrotic debris, later transitioning to an anti‑inflammatory phenotype that supports repair and remodeling [6]. Both pro-inflammatory and anti-inflammatory macrophages secrete cytokines that regulate satellite cell proliferation and differentiation [7]. Studies demonstrate that the depletion of macrophages during tissue repair process markedly impairs the regeneration of skeletal muscle [8].

The cannabinoid type 2 receptor(CB2R), a G protein-coupled receptor mainly expressed in peripheral immune cells and organs [9], is a key modulator of inflammatory responses [10]. CB2R activation alleviates tissue damage in models like acute liver injury [11] and sepsis-induced brain inflammation [12] by curbing macrophage activation and cytokine release. Our previous studies have found that CB2R is highly and dynamically expressed in macrophages during muscle repair [13] and that its loss disrupts the M1‑to‑M2 transition, exacerbates inflammation, and hinders regeneration [14]. However, the precise mechanisms by which CB2R influences macrophage function in muscle repair remain to be elucidated.

Growing attention has focused on pyroptosis, a pro‑inflammatory cell death mediated by the NLRP3 inflammasome and gasdermin D (GSDMD) [15]. Upon activation, NLRP3 recruits caspase‑1, which cleaves pro‑IL‑1β/pro‑IL‑18 into active forms [16] and processes GSDMD to generate its pore‑forming N‑terminal fragment (GSDMD‑NT), leading to cytokine release [17]. Observed chiefly in macrophages [18] and neutrophils [19], pyroptosis plays a dual role: moderate activity aids debris clearance and inflammation initiation, whereas excessive activation exacerbates tissue damage and delays healing [20]. Although pyroptosis inhibition promotes muscle regeneration [21], whether CB2R regulates this process is unknown. The PI3K/AKT/NF‑κB pathway is a central regulator of inflammation, macrophage polarization [22], pyroptosis [23], and myogenic differentiation [24]. Its dysregulation can exacerbate inflammasome activation and cytokine release [25]. We therefore hypothesize that the protective effect of CB2R in skeletal muscle regeneration may be mediated by the regulation of the PI3K/AKT/NF-κB signaling pathway.

In this study, we aim to elucidate the role of CB2R in skeletal muscle regeneration by focusing on its regulatory effects on macrophage pyroptosis. We will define the spatiotemporal pattern of pyroptosis after injury, dissect how CB2R influences it via the PI3K/AKT/NF‑κB pathway, and assess whether CB2R‑dependent modulation of pyroptosis affects myogenic differentiation using a conditioned‑medium assay with peritoneal macrophages and C2C12 myoblasts. Our work bridges cannabinoid signaling, innate immune cell death, and muscle regeneration, offering new therapeutic insights for muscle injury.

## Results

### Macrophages represent the predominant source of NLRP3/GSDMD-mediated pyroptosis during skeletal muscle regeneration

**Fig. 1:**
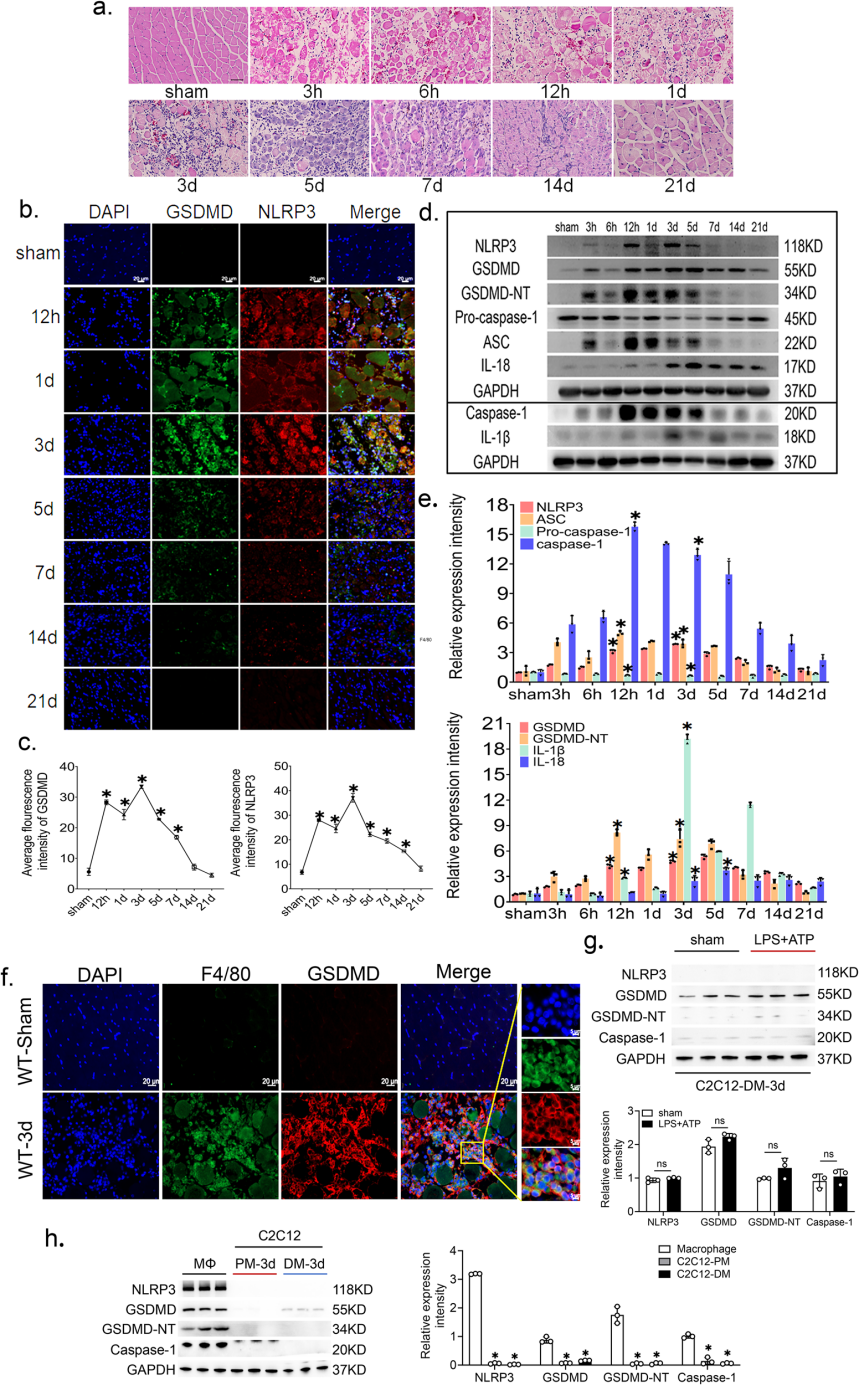
Macrophages as the main cell type undergoing pyroptosis after skeletal muscle injury. **a** Representative images of histomorphology of the process of skeletal muscle injury repair, scale bar = 50 μm. **b** Representative immunofluorescence images of NLRP3 and GSDMD, scale bar = 20 μm. **c** Average fluorescence intensity of NLRP3 and GSDMD, *n* = 3. **d**, **e** Western blot and quantitative analysis of NLRP3 inflammasome, caspase-1, GSDMD, GSDMD-NT, IL-1β and IL-18 proteins from whole muscle tissue lysates, *n* = 3. **f** Representative immuno- fluorescence pictures of F4/80(green) and GSDMD(red) colocalization in the injury area 3 days after skeletal muscle injury, large image scale bar = 20 μm, small image scale bar = 5 μm. Data are represented as mean ± SD, *p < 0.05 versus sham group. **g** Western blot analysis of pyroptosis markers (NLRP3, cleaved caspase-1, and GSDMD-NT) in differentiated C2C12 myotubes treated with or without LPS + ATP. *n* = 3; ns not significant. **h** Representative Western blots and quantification of pyroptosis markers in primary peritoneal macrophages, C2C12, and differentiated C2C12 myotubes under identical LPS + ATP stimulation. *n* = 3, data are represented as mean ± SD; *p < 0.05 versus macrophage group.

H&E staining confirmed successful establishment of the contusion model, showing the typical progression from hemorrhage and inflammation to complete regeneration by day 21 (Fig. 1a). NLRP3/GSDMD co‑staining revealed that pyroptosis occurred predominantly within the first 7 days post‑injury, with strong signal localized to inflammatory cells in the injured area at day 3 (Fig. 1b, c). Western blot analysis showed time‑dependent upregulation of the NLRP3 inflammasome, followed by its effector GSDMD‑NT (peaking at 12 h and 3 d), and the downstream cytokines IL‑1β (day 3) and IL‑18 (day 5) (Fig. 1d, e). Based on these findings, we focused on the 3‑day time point and confirmed abundant macrophage pyroptosis within the injury site (Fig. 1f).

To determine whether macrophages were the primary cell type undergoing pyroptosis, we performed complementary in vitro assays. Differentiated C2C12 myotubes exposed to LPS + ATP showed no significant induction of NLRP3, cleaved caspase‑1, or GSDMD‑NT (Fig. 1g). Moreover, under identical stimulatory conditions, robust NLRP3 inflammasome activation and GSDMD cleavage were observed only in peritoneal macrophages, not in undifferentiated C2C12 myoblasts or differentiated myotubes (Fig. 1h). Together, these data demonstrate that macrophages are the predominant cell type executing NLRP3/GSDMD‑mediated pyroptosis in the injured skeletal muscle microenvironment.

### CB2R knockout aggravated macrophages pyroptosis after skeletal muscle injury

**Fig. 2:**
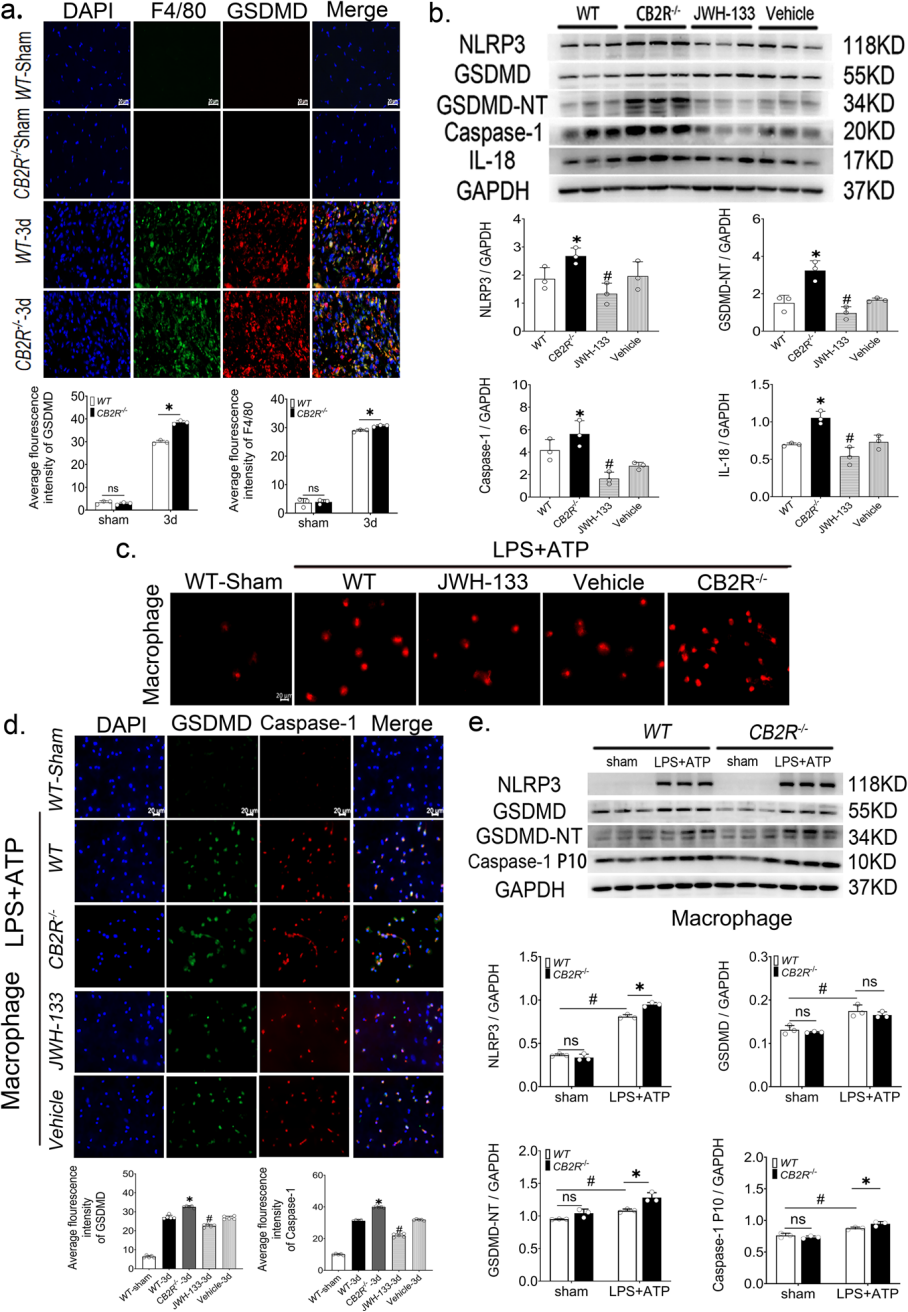
CB2R knockout exacerbates macrophage pyroptosis after skeletal muscle injury. **a** The average fluorescence intensity of F4/80 and GSDMD, scale bar = 20μm, *n* = 3, *p < 0.05 vs. WT group. **b** Western blot and quantitative analysis of NLRP3, GSDMD, GSDMD-NT, caspase-1 and IL-18 in each group 3 days after skeletal muscle injury, *n* = 3, *p < 0.05 vs. WT group, *#p* < 0.05 vs. Vehicle group. **c** PI pictures of peritoneal macrophages treated with LPS + ATP. **d** Immunofluorescence of caspase-1(red) and GSDMD (green), blue channel is DAPI stained nuclei, scale bar = 20 μm, *n* = 5, *p < 0.05 vs. WT-3d group, #*p* < 0.05 vs. Vehicle group. **e** Western blot and quantitative analysis of NLRP3, GSDMD, GSDMD-NT and caspase-1, *n* = 3, *p < 0.05 vs. WT group, #*p* < 0.05 vs. sham group. Data are represented as mean ± SD. ns no significance.

Our findings demonstrate that CB2R expression undergoes a dynamic temporal change following skeletal muscle injury, characterized by an initial upregulation followed by a decline(Supplementary Fig. 1E). Notably, CB2R expression is markedly elevated in macrophages at day 3 post-injury (Supplementary Fig. 1F). To investigate the correlation between CB2R and macrophage pyroptosis, we used CB2R gene knockout mice. The co-staining immunofluorescence of F4/80 and GSDMD proved that CB2R knockout exacerbated GSDMD expression in macrophages and intensified macrophage pyroptosis (Fig.2a). Additionally, compared to wild-type mice, CB2R knockout led to a increased number of inflammatory cells (Supplementary Fig. 2A) in the damaged area and also led to increased expression of NLRP3, caspase-1, GSDMD-NT, IL-1β and IL-18, whereas CB2R activation yielded opposite outcomes(Supplementary Fig. 2B, Fig. 2b). Subsequently, PI experiments and co-staining immunofluorescence of GSDMD and caspase-1 in primary peritoneal macrophages in vitro revealed that CB2R knockout exacerbated macrophage pyroptosis induced by LPS + ATP (Supplementary Fig. 2C, Fig. 2c, d). Western blotting results further demonstrated that LPS + ATP treatment in CB2R knockout macrophages increased protein expression levels of NLRP3, caspase-1, and GSDMD-NT compared to the WT group(Fig. 2e).

### CB2R knockout promoted the activation of PI3K/AKT/NF-κB pathway

**Fig. 3:**
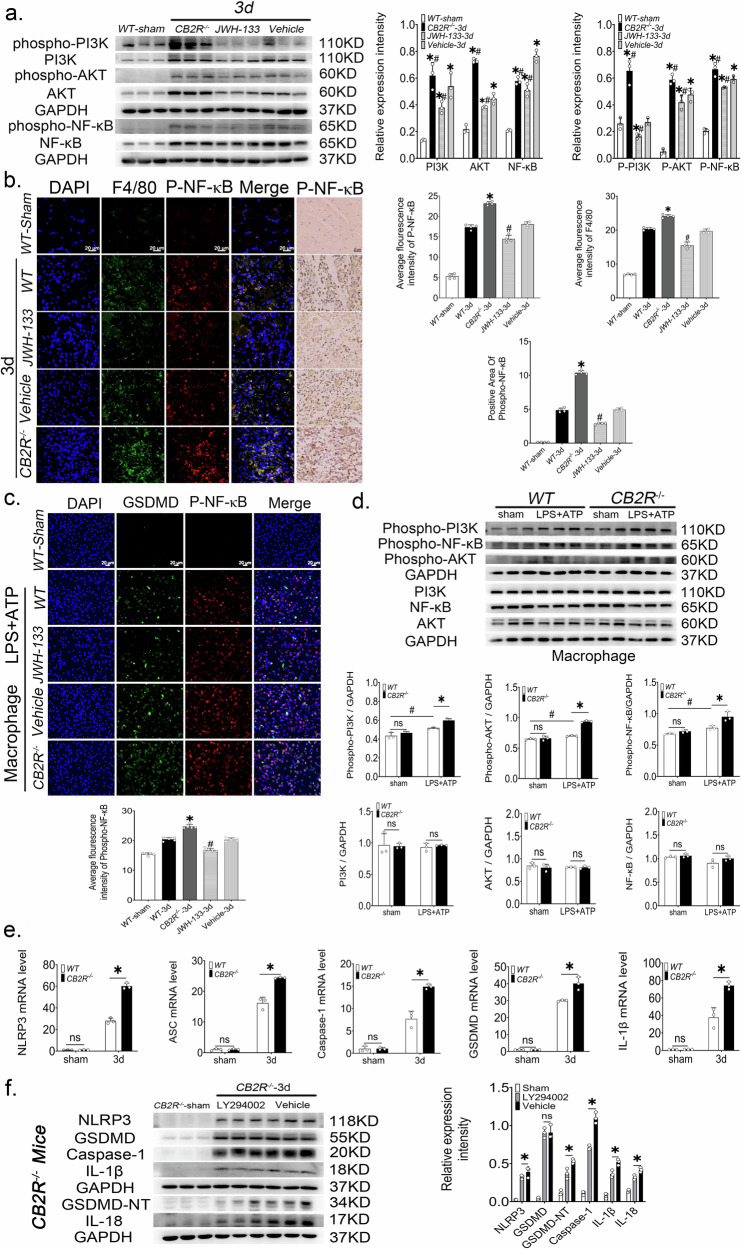
CB2R regulates macrophage pyroptosis through the PI3K/AKT/NF-κB pathway. **a** Western blot and quantitative analysis of PI3K, phospho-PI3K, AKT, phospho-AKT, NF-κB and phospho-NF-κB from whole muscle tissue lysates, *n* = 3, *p < 0.05 vs. sham group, #*p* < 0.05 vs. Vehicle group. **b** The positive area of phospho-NF-κB expression and the average fluorescence intensity of F4/80 and phospho-NF-κB in the damaged area, *n* = 4, *p < 0.05 vs. WT-3d group, #*p* < 0.05 vs. Vehicle-3d group. **c** Relative expression of phospho-NF-κB in peritoneal macrophages treated with LPS + ATP. *n* = 5, *p < 0.05 vs. WT group, #*p* < 0.05 vs. Vehicle group. **d** PI3K, phospho-PI3K, AKT, phospho-AKT, NF-κB and phospho-NF-κB protein levels in peritoneal macrophages of WT mice and *Cb2r*^*−/−*^ mice after stimulation with LPS + ATP, *n* = 3, *p < 0.05 vs. WT group, #*p* < 0.05 vs. sham group. Data are represented as mean ± SD. **e** NLRP3, ASC, caspase-1, GSDMD and IL-1β mRNA expression levels, *n* = 3, *p < 0.05 vs. WT group. **f** The protein levels of NLRP3, GSDMD, GSDMD-NT, caspase-1, IL-18 and IL-1β from whole muscle tissue lysates of *Cb2r*^*−/−*^ mice at 3 days post-injury treated with the PI3K inhibitor LY294002 or vehicle, *n* = 3, *p < 0.05 vs. Vehicle group. Data are represented as mean ± SD. ns no significance.

To explore the mechanism by which CB2R regulates pyroptosis, we examined the PI3K/AKT/NF‑κB pathway. Western blot analysis showed that skeletal muscle injury increased phospho‑PI3K, phospho‑AKT, and phospho‑NF‑κB levels, which were further elevated in CB2R‑deficient mice. In contrast, CB2R activation significantly suppressed NF‑κB phosphorylation (Fig. 3a). Immunohistochemistry revealed no significant difference in phospho‑NF‑κB between vehicle‑ and WT‑injury groups at day 3 (Fig. 3b). In vitro, LPS + ATP‑stimulated peritoneal macrophages exhibited higher phospho‑PI3K, phospho‑AKT, and phospho‑NF‑κB expression than unstimulated controls. Moreover, CB2R knockout further increased phospho‑NF‑κB levels, indicating enhanced activation of this pathway (Fig. 3c, d).

Given the central role of NF‑κB in transcriptional regulation [26], we also assessed mRNA levels of key pyroptosis‑related molecules. qRT‑PCR demonstrated significant upregulation of *Nlrp3*, *Asc*, *Caspase‑1*, *Gsdmd*, and *Il‑1β* after injury, consistent with elevated expression of pyroptosis‑associated proteins (Fig. 3e).

### CB2R regulates macrophage pyroptosis through the PI3K/AKT/NF-κB pathway

Intraperitoneal administration of the PI3K inhibitor LY294002 (10 mg/kg daily for 3 days) effectively suppressed PI3K/AKT/NF‑κB activation in *Cb2r*^*−/−*^ mice (Supplementary Fig. 2D). Consequently, the levels of pyroptosis‑related proteins (NLRP3, caspase‑1, GSDMD‑NT) and cytokines (IL‑1β, IL‑18) were significantly reduced compared with vehicle‑treated *Cb2r*^*−/−*^ mice(Fig. 3f). These results demonstrate that pharmacological blockade of the PI3K/AKT/NF‑κB axis substantially alleviates pyroptosis in CB2R‑deficient mice, thereby confirming that CB2R restrains macrophage pyroptosis through modulation of this signaling pathway.

### Inhibition of pyroptosis promotes muscle regeneration after injury

**Fig. 4:**
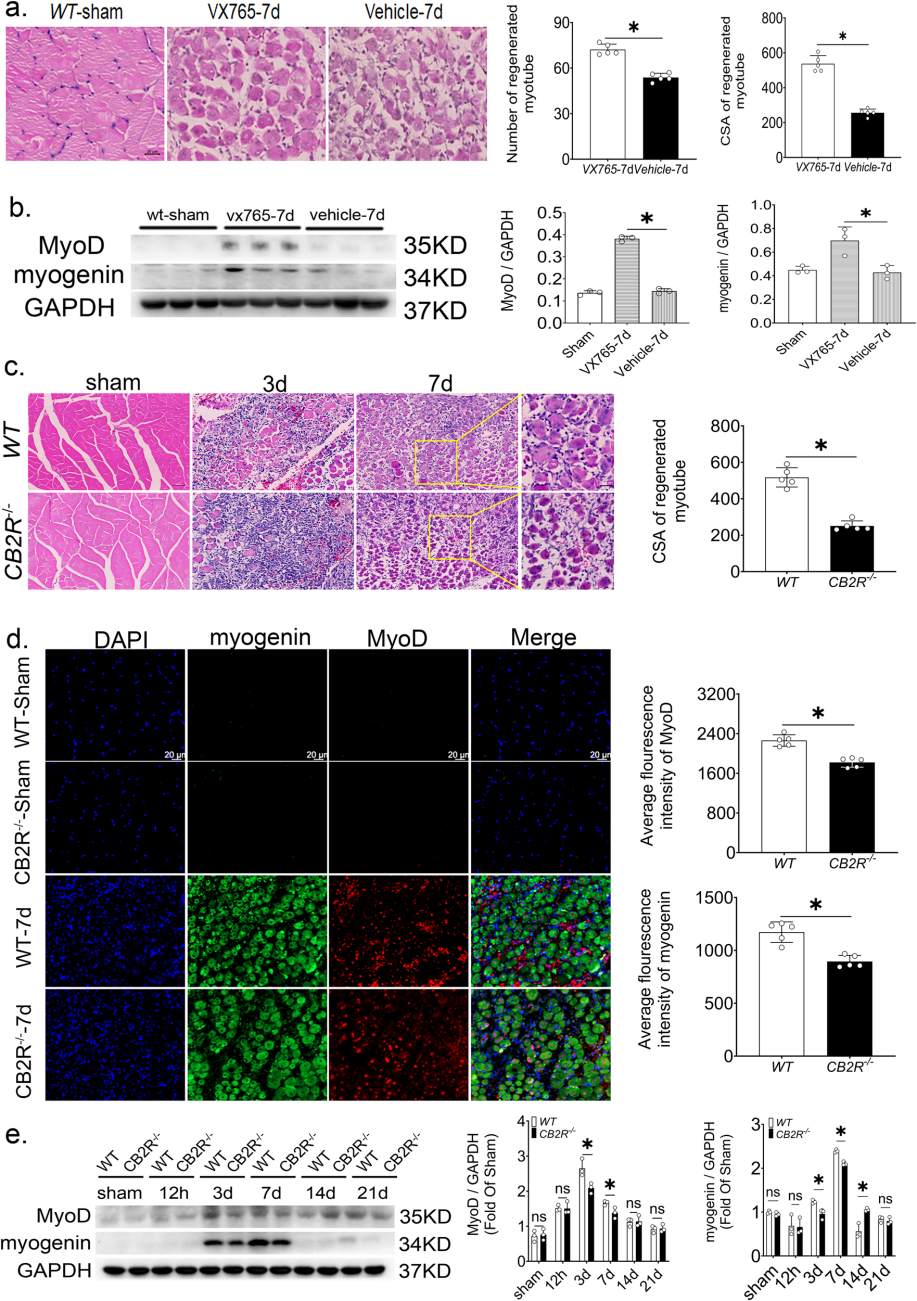
Inhibiting pyroptosis can promote skeletal muscle regeneration after injury, however, CB2R knockout impeded skeletal muscle regeneration after injury. **a** Cross-sectional area and number of regenerated muscle cells 7 days after skeletal muscle injury, *n* = 5, *p < 0.05 vs. Vehicle group. **b** MyoD and Myogenin protein expression levels from whole muscle tissue lysates at 7 days post-injury in mice treated with VX765 compared to the vehicle-treated group, *n* = 3, *p < 0.05 vs. Vehicle group. **c** Statistical diagram of diameter and cross-sectional area of regenerated muscle cells, *n* = 5, *p < 0.05 WT vs. *Cb2r*^*−/−*^ group, large picture scale bar = 50 μm, small picture scale bar = 20 μm. **d** Expression levels of MyoD and Myogenin, scale bar = 20 μm, *n* = 5, *p < 0.05 WT vs. *Cb2r*^*−/−*^ group. **e** MyoD and Myogenin protein levels after injury in WT and *Cb2r*^*−/−*^ mice, *n* = 3, *p < 0.05 WT vs. *Cb2r*^*−/−*^ group. Data are represented as mean ± SD. ns no significance.

To assess the impact of pyroptosis on regeneration, the caspase‑1 inhibitor VX765 (100 mg/kg) was administered i.p. for 7 days post‑injury. At day 7, VX765‑treated mice showed significantly larger regenerated myofiber cross‑sectional area (CSA) and increased myofiber numbers compared with the vehicle group (Fig. 4a). Western blot analysis further revealed elevated protein levels of the myogenic markers MyoD and Myogenin in the VX765 group (Fig. 4b), indicating that suppression of pyroptosis enhances the regenerative myogenic program.

### CB2R knockout hindered skeletal muscle regeneration after injury

Next, the impact of CB2R knockout on skeletal muscle regeneration was evaluated after injury. The diameter of newly regenerated fibers and CSA were measured in *Cb2r*^*−/−*^ mice 7 days post-injury. Results showed a significant decrease in the diameter and CSA of muscle fibers compared to WT mice, implying that CB2R knockout hinder muscle cell regeneration(Fig. 4c). Conversely, CB2R agonist JWH‑133 increased myofiber CSA and myogenic markers, promoting regeneration (Supplementary Fig. 2E). Immunofluorescence and western blot analysis revealed lower expression levels of MyoD and Myogenin, markers associated with muscle regeneration, in *Cb2r*^*−/−*^ mice compared to WT mice at the same time point (Fig. 4d, e).

### Double knockout of NLRP3 and CB2R reversed the impeded post-injury muscle regeneration caused by CB2R knockout

**Fig. 5:**
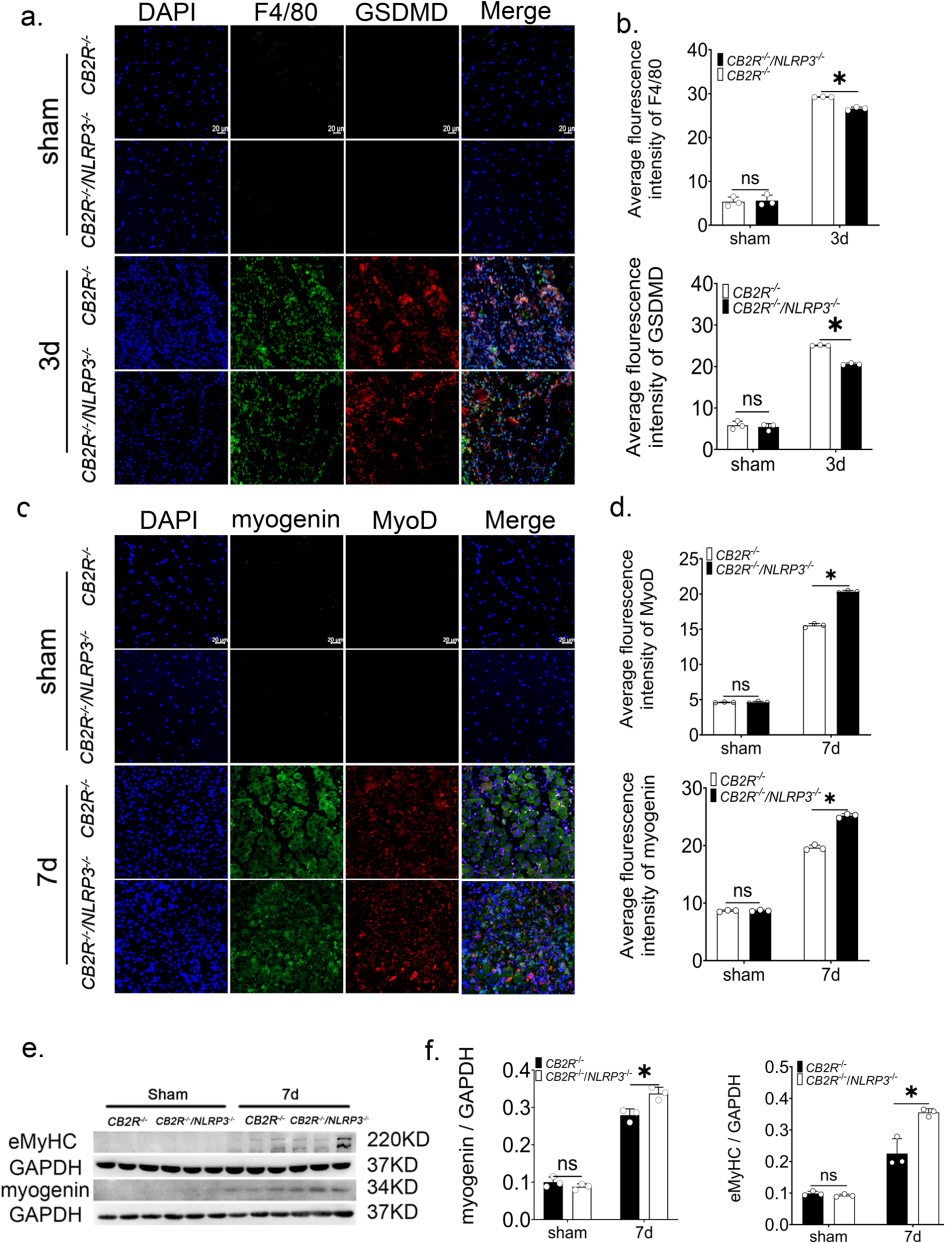
Further knocking out NLRP3 on the basis of CB2R knockout can alleviate the limitation of skeletal muscle regeneration caused by CB2R knockout. **a** Representative images of GSDMD (red) and F4/80 (green) in *Cb2r*^*−/−*^ mice and *Cb2r*^*−/−*^*/Nlrp3*^*−/−*^ mice, scale bar = 20 μm. **b** GSDMD expression levels in macrophages, *n* = 3, *p < 0.05 *Cb2r*^*−/−*^ vs. *Cb2r*^*−/−*^*/Nlrp3*^*−/−*^ group. **c**, **d** Expression levels of MyoD and Myogenin in regenerated muscle cells of *Cb2r*^*−/−*^*/Nlrp3*^*−/−*^mice and *Cb2r*^*−/−*^*/Nlrp3*^*−/−*^ mice, scale bar = 20 μm, *n* = 3, *p < 0.05 *Cb2r*^*−/−*^ vs. *Cb2r*^*−/−*^*/Nlrp3*^*−/−*^group. **e**, **f** Western blot and quantitative analysis of eMyHC and Myogenin protein expression levels from whole muscle tissue lysates of mice at 7 days post-injury compared to sham group, *n* = 3, *p < 0.05 *Cb2r*^*−/−*^ vs. *Cb2r*^*−/−*^*/Nlrp3*^*−/−*^ group. Data are represented as mean ± SD. ns no significance.

To genetically dissect the relationship between CB2R, pyroptosis and regeneration, we employed *Cb2r*^*−/−*^ and *Cb2r*^*−/−*^*/Nlrp3*^*−/−*^ mice. At 3 days post‑injury, immuno- fluorescence co‑staining of F4/80 and GSDMD showed that macrophage‑specific GSDMD expression—and thus pyroptosis—was markedly reduced in *Cb2r*^*−/−*^*/Nlrp3*^*−/−*^ versus *Cb2r*^*−/−*^ mice (Fig. 5a, b). By day 7, the *Cb2r*^*−/−*^*/Nlrp3*^*−/−*^ mice exhibited significantly larger regenerating myofiber cross‑sectional area (Supplementary Fig. 2F) and higher expression of the myogenic regulators MyoD and Myogenin (Fig. 5c, d). Western blot analysis confirmed that protein levels of eMyHC and Myogenin were elevated in *Cb2r*^*−/−*^*/Nlrp3*^*−/−*^ mice compared with *Cb2r*^*−/−*^ mice (Fig. 5e, f).

### CB2R knockout macrophages impeded C2C12 cell differentiation

**Fig. 6:**
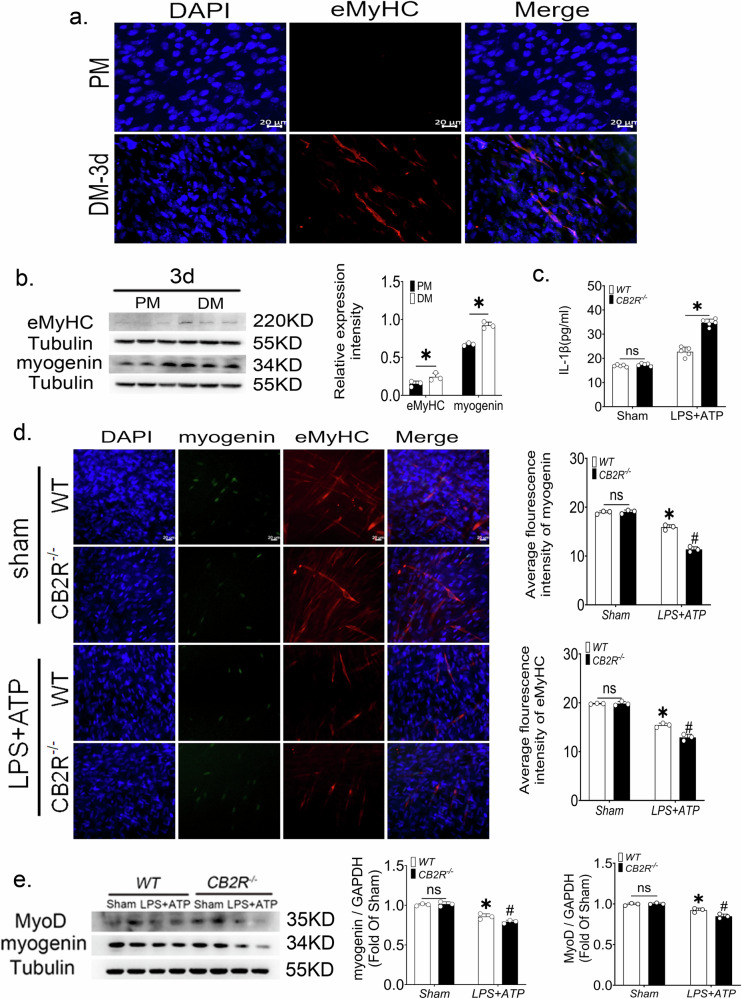
CB2R knockout macrophages impede the differentiation of C2C12 cells. **a** Representative pictures of immunofluorescence of eMyHC, scale bar = 20 μm. **b** eMyHC and Myogenin protein expression levels of C2C12 cells, *n* = 3, *p < 0.05 PM-3d vs. DM-3d group. **c** The release of IL-1β from macrophages after LPS + ATP treatment, *n* = 5, *p < 0.05 *Cb2r*^*−/−*^ vs. WT group. **d** Expression of Myogenin and eMyHC in C2C12 cells after treatment with peritoneal macrophage-conditioned medium, scale bar = 20 μm, *n* = 3, *p < 0.05 sham vs. LPS + ATP group, #*p* < 0.05 WT vs. *Cb2r*^*−/−*^ group. **e** MyoD and Myogenin protein expression levels in C2C12 cells in different treatment groups, *n* = 3, *p < 0.05 sham vs. LPS + ATP group, #*p* < 0.05 WT vs. *Cb2r*^*−/−*^ group. Data are represented as mean ± SD. ns no significance.

To test whether macrophage‑secreted factors influence myogenesis, C2C12 cells were treated with conditioned medium (CM) from peritoneal macrophages. While minimal myotube formation occurred in proliferation medium, robust differentiation—marked by elevated eMyHC and Myogenin expression—was observed after 3 days in differentiation medium (Fig. 6a, b).

CM from LPS + ATP‑stimulated macrophages contained elevated IL‑1β, and this effect was further amplified when macrophages lacked CB2R (Fig. 6c). IL‑1β secretion was significantly reduced in CM from *Cb2r*^*−/−*^/*Nlrp3*^−/−^ versus *Cb2r*^*−/−*^ macrophages confirming NLRP3‑dependence (Supplementary Fig. 3A). Conversely, the expressions of MyoD and Myogenin in C2C12 cells were significantly suppressed after 3 days with conditioned differentiation medium from *Cb2r*^*−/−*^ macrophages compared to WT cells, proving that *Cb2r*^*−/−*^ macrophages significantly impeded the differentiation of C2C12 cells (Fig. 6d, e). This inhibition was substantially rescued when C2C12 cells were treated with CM from *Cb2r*^*−/−*^/*Nlrp3*^−/−^ macrophages, as assessed by eMyHC expression (Supplementary Fig. 3B, C). Collectively, these results demonstrate that CB2R deficiency in macrophages enhances NLRP3‑dependent IL‑1β release, which in turn impairs C2C12 myogenic differentiation.

## Discussion

Skeletal muscle regeneration following injury involves a finely tuned interplay between immune responses and myogenic programs [27]. Among the immune mediators, macrophages play a central role by orchestrating both the inflammatory and resolution phases of regeneration [5]. Our study identifies macrophage pyroptosis—a highly inflammatory form of programmed cell death [28]—as a critical modulator of this process, a concept further solidified by recent work highlighting its relevance in muscle pathology and repair [29, 30]. At day 3 post-injury, we observed significant upregulation of key pyroptosis-related proteins (NLRP3, cleaved Caspase-1, GSDMD-NT) and inflammatory cytokines (IL-1β, IL-18). This molecular signature correlated with impaired regenerative outcomes, including smaller regenerating myofibers and downregulation of myogenic markers (MyoD, Myogenin, eMyHC), consistent with reports linking sustained inflammation to compromised myogenesis [31]. Notably, pharmacological inhibition of Caspase-1 with VX765 attenuated these deficits, underscoring pyroptosis as a tractable contributor to the post-injury milieu and aligning with studies that implicate inflammasome activation in chronic muscle damage [32]. While the role of pyroptosis in inflammatory diseases is increasingly recognized [33], its regulation and functional impact within the dynamic context of acute skeletal muscle repair remained poorly defined. Our findings address this gap by establishing pyroptosis as a pivotal, early inflammatory event in muscle regeneration.

A novel aspect of our study is the delineation of CB2R as a key regulator of both macrophage pyroptosis and the immune microenvironment. While CB2R has been implicated in modulating immune responses in various inflammatory contexts [34], and our prior work established its cell-autonomous role in promoting myogenic survival and differentiation [14], its non‑cell‑autonomous role in orchestrating repair via macrophages was unclear. Here, we demonstrate that CB2R deficiency exacerbates NLRP3 inflammasome activation, IL‑1β/IL‑18 release, and myofiber regeneration defects. This regulatory function likely involves nuanced control of macrophage heterogeneity. Macrophages exist along a continuum from pro‑inflammatory (M1) to pro‑reparative (M2) phenotypes [35], and growing evidence indicates a propensity for pyroptosis in M1 macrophages [36, 37]—potentially creating a feed‑forward loop that sustains inflammation and impedes transition to a repair‑permissive state. The phenotype of CB2R‑deficient mice, characterized by enhanced pyroptosis and impaired repair, aligns with the established role of CB2R in promoting the M1‑to‑M2 transition [14]. Together, these observations position CB2R as a key determinant of macrophage function and fate during muscle regeneration.

Mechanistically, we show that CB2R restrains pyroptosis via the PI3K/AKT/NF-κB axis—a well‑established pathway governing inflammatory gene expression and cell survival [38]. In CB2R‑deficient peritoneal macrophages, inflammatory stimulation hyperactivated this axis, elevating NLRP3 and other pyroptosis‑related proteins. Pharmacologic PI3K inhibition reduced AKT/NF‑κB activation and markedly suppressed pyroptosis. Although PI3K/AKT has been linked to CB2R signaling in other contexts [39], and NF‑κB is a recognized master regulator of NLRP3 transcription [40], our work is the first to integrate these components into a coherent CB2R–PI3K/AKT/NF-κB–NLRP3 pathway that controls macrophage pyroptosis within the skeletal muscle niche. Given the dual role of NF‑κB in driving both inflammatory responses and satellite‑cell activity [41], CB2R‑mediated tuning of this axis represents a crucial intersection where immune regulation meets muscle regeneration.

To functionally link CB2R to NLRP3‑dependent pyroptosis in vivo, we performed a genetic rescue. Deleting *Nlrp3* in the *Cb2r*^−/−^ background restored myogenic gene expression and improved myofiber regeneration, directly establishing the NLRP3 inflammasome as a downstream effector of CB2R signaling. These data confirm that CB2R promotes repair largely by restraining inflammasome activity. In vitro, macrophage‑conditioned medium revealed how altered immune signaling impairs regeneration non‑cell‑autonomously: CB2R‑deficient macrophages, through heightened IL‑1β secretion, directly inhibited C2C12 myoblast differentiation. This underscores the critical role of the immune microenvironment in shaping regenerative outcomes and reinforces the principle that inflammation must be tightly controlled in space and time for effective tissue repair [42].

In conclusion, our study provides the first mechanistic link between CB2R signaling and the suppression of NLRP3 inflammasome-mediated pyroptosis in macrophages during muscle regeneration. The functional integration of these components into a CB2R–PI3K/AKT/NF-κB–NLRP3 axis represents a significant conceptual advance in understanding immune-regenerative crosstalk. This finding not only elucidates a novel regulatory pathway but also offers a compelling therapeutic rationale. Given the paucity of clinical options for improving muscle repair, targeting this axis may present a promising strategy to accelerate recovery, particularly in contexts of dysregulated inflammation [10, 43]. While our systemic knockout and in vitro models provide robust evidence, future studies employing myeloid-specific conditional knockouts will be valuable to precisely define the macrophage-autonomous functions of this axis within the complex muscle niche.

## Materials and methods

### Animal and grouping

Male C57BL/6J mice (8–10 weeks, 25 ± 2 g) were used. *Cb2r*^−/−^ mice were from Jackson Laboratory; *Cb2r*^−/−^/*Nlrp3*^−/−^ mice were from the Animal Experimental Center of China Medical University. Genotypes were confirmed by PCR of toe-DNA (Supplementary Fig. 1A, B). After confirmation, mice were randomly allocated to groups. WT mice were divided into: sham, injury, injury + JWH‑133, injury + VX765, and injury + vehicle. *Cb2r*^−/−^mice were divided into: sham, injury, injury + LY294002, and injury + vehicle. *Cb2r*^−/−^/*Nlrp3*^−/−^ mice were divided into sham and injury groups. The sample size for each experimental group (*n* = 3–5 mice) was determined based on established standards in skeletal muscle regeneration research and our laboratory’s prior experience with the contusion model [44, 45], which has provided sufficient power while adhering to reduction principles. Injury groups were sampled at 3 and 7 days post‑contusion. Drugs (JWH‑133, 10 mg/kg [46]; VX765, 100 mg/kg [47]; LY294002, 10 mg/kg [48]) or vehicle (5% DMSO, 40% PEG300, 5% Tween‑80, 50% saline) were administered i.p. once daily for 3 or 7 days as indicated.

### Animal model of skeletal muscle injury

The model of skeletal muscle injury was established by the method reported previously by our team [13]. Briefly, mice were anesthetized (2% sodium pentobarbital), and hair on the right hindlimb was removed. The following day, deep anesthesia was induced with isoflurane. The limb was fixed in a 90° position, and a blunt percussion device (speed 3 m/s, deformation 5 mm, strike time 50 ms, strike area 19.625 mm²) was used to induce muscle injury without fracture. Mice remained anesthetized throughout the procedure. Sham mice underwent anesthesia and hair removal only (Supplementary Fig. 1C). Post‑injury, mice were singly housed with sterile food/water. Only animals that completed the protocol without complications (e.g., fracture, severe distress, or infection) and had confirmed correct genotype were included in the final analysis.

### Hematoxylin-eosin (H&E) staining

Gastrocnemius muscle samples were fixed in a 4% paraformaldehyde solution, embedded in paraffin. 5μm-thick sections were prepared for H&E staining, and imaged using a Zeiss Axio Scan.Z1 confocal microscope system(Zeiss, Jena, Germany).

### Immunohistochemistry

Paraffin sections of gastrocnemius muscle were deparaffinized, rehydrated, and subjected to antigen retrieval in citrate buffer. Endogenous peroxidase activity was quenched with 3% H₂O₂, followed by blocking with normal goat serum. Sections were incubated overnight at 4 °C with primary antibodies against caspase‑1 (1:200, sc56036, Santa Cruz), IL‑1β (1:200, YT5201, Immunoway), and phospho‑NF‑κB (1:200, 4060, CST). After incubation with a biotin‑conjugated secondary antibody, signals were developed using DAB, and nuclei were counterstained with hematoxylin. Images were acquired with a Zeiss Axio Scan.Z1 system.

### Immunofluorescence

Paraffin-embedded skeletal muscle sections were dewaxed, dehydrated, and subjected to antigen retrieval in 0.01 mmol/L citrate buffer (pH 6.0). Cells (peritoneal macrophages or C2C12 myoblasts) were seeded on glass coverslips, fixed with 4% paraformaldehyde, and permeabilized with 0.1% Triton X-100. All samples were blocked with donkey serum (5% for tissues, 8% for cells) for 2 h at room temperature, followed by incubation with primary antibodies overnight at 4 °C. The following antibodies were used: F4/80 (1:400, 28463-1-AP, Proteintech), GSDMD (1:200, sc393656, Santa Cruz), NLRP3 (1:200, 15101S, CST), CD86 (1:200, sc-28347, Santa Cruz), CD206 (1:200, sc58986, Santa Cruz), caspase-1 (1:200, 22915-1-AP, Proteintech), eMyHC (1:50, F1.652, DSHB), Myogenin (1:200, ab124800, Abcam), and phospho-NF-κB (1:200, 4060, CST). After washing, samples were incubated with secondary antibodies for 2 h, counterstained with DAPI, and mounted with aqueous mounting medium. Images were acquired using a Zeiss Axio Scan.Z1 confocal microscope.

### Western blot

Western blotting was performed as follows. Proteins were extracted from pulverized gastrocnemius muscle or cultured cells (peritoneal macrophages, C2C12) using cold RIPA lysis buffer supplemented with PMSF and phosphatase inhibitors, and concentrations were determined by BCA assay. Equal amounts of protein (15 μg) were separated by SDS‑PAGE and transferred to PVDF membranes. After blocking with 5% BSA or 8% skim milk, membranes were incubated overnight at 4 °C with primary antibodies against: NLRP3 (1:1000, 1510S, CST), ASC (1:1000, sc514414, Santa Cruz), Pro‑caspase‑1 (1:1000, ab1872, Abcam), caspase‑1 (1:1000, sc56036, Santa Cruz), GSDMD (1:1000, ab209548, Abcam), GSDMD‑NT (1:1000, sc393656, Santa Cruz), IL‑18 (1:1000, ab191860, Abcam), IL‑1β (1:1000, abs120224, Absin), phospho‑PI3K (1:1000, 341468, ZENBIO), PI3K (1:1000, 251221, ZENBIO), phospho‑AKT (1:1000, 4060, CST), AKT (1:1000, 4691, CST), phospho‑NF‑κB (1:1000, 3033, CST), NF‑κB (1:1000, 10745, Proteintech), and GAPDH (1:3000, 60004, Proteintech). After washing, membranes were incubated with HRP‑conjugated secondary antibody for 2 h at room temperature. Signals were developed using an ECL kit and imaged; band intensities were quantified with ImageJ.

### qRT-PCR assays

Total RNA was extracted from gastrocnemius muscle using TRIzol reagent (Thermo Fisher Scientific) and quantified spectrophotometrically. After adjusting the concentration, 250 ng RNA was reverse‑transcribed into cDNA using the PrimeScript RT kit (TaKaRa). Quantitative PCR was performed in 10 μL reactions containing TB Green Premix Ex Taq II (TaKaRa), 0.4 μM each forward/reverse primer, and 2 μL cDNA. Amplification was carried out on a Roche real‑time PCR system. Relative mRNA expression of *Nlrp3*, *asc*, *caspase‑1*, *gsdmd*, and *IL‑1β* was calculated using the 2^(−ΔΔCt)^ method with *Gapdh* as the endogenous control. Primer sequences are listed in Supplementary Table 1. All reactions were run in triplicate.

### Isolation of peritoneal macrophages

Peritoneal macrophages were isolated from 6-8 weeks old adult WT and *Cb2r*^*−/−*^ mice following established protocols [49]. The cells were cultured at 37 °C in a 5% CO_2_ environment for two hours. Non-adherent cells were removed using the differential adherence method, and the adherent cells were further cultured in DMEM medium(Supplementary Fig. 1D).

### Induced pyroptosis of peritoneal macrophages

Adherent macrophages were pretreated with the CB2R agonist JWH‑133 (10 μM) or DMSO (vehicle) for 24 h, washed, and then stimulated with LPS (500 ng/mL, 24 h) followed by ATP (5 mM, 1 h). After washing, cells were cultured in serum‑free DMEM for 24 h to generate conditioned medium (CM), which was collected for ELISA and C2C12 cell treatment.

### Propidium Iodide staining

Cell membrane permeability was assessed by propidium iodide (PI) staining. Peritoneal macrophages cultured in 24‑well plates were pretreated with 10 μM JWH‑133 or DMSO (control) for 24 h, washed with PBS, and then stimulated with LPS + ATP. Cells were incubated with PI (2 μg/mL) for 15 min at room temperature and immediately imaged using a Zeiss Axio Scan.Z1 confocal microscope.

### ELISA

The collected macrophage conditioned medium was centrifuged at 2000 × *g* for 10 min, and the supernatant was isolated. The concentration of IL-1β in the conditioned medium of each group was measured according to the procedures provided by the IL-1β detection kit(Elabscience, Wuhan, China).

### C2C12 cell culture and treatment with macrophage-conditioned medium

C2C12 cells (Procell, Wuhan, China) were STR‑authenticated and mycoplasma‑free. Cells were maintained in high‑glucose DMEM with 10% FBS and 1% antibiotics at 37 °C/5% CO₂. Differentiation was induced at ~90% confluence by switching to DMEM containing 2% horse serum (differentiation medium, DM) and monitored up to 5 days.

To assess the effect of macrophage‑secreted factors, confluent C2C12 cells were cultured for 3 days in conditioned differentiation medium (25% macrophage‑conditioned medium + 75% fresh DM), with medium replaced every 24 h. Cells were then harvested and analyzed for differentiation markers by Western blot and immunofluorescence.

### Morphometric and statistical analyses

Histological sections stained with H&E were analyzed to determine muscle fiber cross-sectional area and diameter using ImageJ software, as previously described [50]. IF images were quantified for positive area (percentage of pixels above a set intensity threshold) and mean fluorescence intensity (average intensity within a defined ROI) using ImageJ by an observer blinded to group allocation. Data were obtained from at least three mice per group, with five random fields analyzed per animal, and are presented as mean ± SD from a minimum of three independent experiments.

Statistical analyses were performed with Prism 9.0 (GraphPad Software). Normality was assessed using the Shapiro–Wilk test, and homogeneity of variance was evaluated with the *F*‑test (for *t*‑tests) or Brown–Forsythe and Bartlett’s tests (for ANOVA). Comparisons between two groups were made by unpaired Student’s *t* test. Comparisons among three or more groups were analyzed by one‑way ANOVA followed by Tukey’s HSD post‑hoc test for pairwise comparisons. A *p‑*value < 0.05 was considered statistically significant.

## Supplementary information

SUPPLEMENTAL MATERIAL

Original western blots

## Data Availability

All datasets generated during or analyzed during the current study are available from the corresponding author upon reasonable request.

## References

[1] Chen N, Fong DYT, Wong JYH. Secular trends in musculoskeletal rehabilitation needs in 191 countries and territories from 1990 to 2019. JAMA Netw Open. 2022;5:e2144198 10.1001/jamanetworkopen.2021.44198. 35044468 10.1001/jamanetworkopen.2021.44198 PMC8771302

[2] Beiner JM, Jokl P. Muscle contusion injuries: current treatment options. J Am Acad Orthop Surg. 2001;9:227–37. 11476532 10.5435/00124635-200107000-00002

[3] Ziemkiewicz N, Hilliard G, Pullen NA, Garg K. The role of innate and adaptive immune cells in skeletal muscle regeneration. Int J Mol Sci. 2021;22. 10.3390/ijms22063265. 10.3390/ijms22063265 PMC8005179 33806895

[4] Dumont NA, Bentzinger CF, Sincennes MC, Rudnicki MA. Satellite cells and skeletal muscle regeneration. Compr Physiol. 2015;5:1027–59. 10.1002/cphy.c140068. 26140708 10.1002/cphy.c140068

[5] Chazaud B. Inflammation and skeletal muscle regeneration: leave it to the macrophages!. Trends Immunol. 2020;41:481–92. 10.1016/j.it.2020.04.006. 32362490 10.1016/j.it.2020.04.006

[6] Southerland KW, Xu Y, Peters DT, Lin X, Wei X, Xiang Y, et al. Skeletal muscle regeneration failure in ischemic-damaged limbs is associated with pro-inflammatory macrophages and premature differentiation of satellite cells. Genome Med. 2023;15:95. 10.1186/s13073-023-01250-y. 37950327 10.1186/s13073-023-01250-y PMC10636829

[7] Shang M, Cappellesso F, Amorim R, Serneels J, Virga F, Eelen G, et al. Macrophage-derived glutamine boosts satellite cells and muscle regeneration. Nature. 2020;587:626–31. 10.1038/s41586-020-2857-9. 33116312 10.1038/s41586-020-2857-9 PMC7116844

[8] Nawaz A, Bilal M, Fujisaka S, Kado T, Aslam MR, Ahmed S, et al. Depletion of CD206(+) M2-like macrophages induces fibro-adipogenic progenitors activation and muscle regeneration. Nat Commun. 2022;13:7058. 10.1038/s41467-022-34191-y. 36411280 10.1038/s41467-022-34191-y PMC9678897

[9] Fulmer ML, Thewke DP. The endocannabinoid system and heart disease: the role of cannabinoid receptor type 2. Cardiovasc Hematol Disord Drug Targets. 2018;18:34–51. 10.2174/1871529X18666180206161457. 29412125 10.2174/1871529X18666180206161457 PMC6020134

[10] Li J, Wang H, Liu D, Li X, He L, Pan J, et al. CB2R activation ameliorates late adolescent chronic alcohol exposure-induced anxiety-like behaviors during withdrawal by preventing morphological changes and suppressing NLRP3 inflammasome activation in prefrontal cortex microglia in mice. Brain Behav Immun. 2023;110:60–79. 10.1016/j.bbi.2023.02.001. 36754245 10.1016/j.bbi.2023.02.001

[11] Cai SL, Fan XG, Wu J, Wang Y, Hu XW, Pei SY, et al. CB2R agonist GW405833 alleviates acute liver failure in mice via inhibiting HIF-1alpha-mediated reprogramming of glycometabolism and macrophage proliferation. Acta Pharm Sin. 2023;44:1391–403. 10.1038/s41401-022-01037-8. 10.1038/s41401-022-01037-8 PMC10310807 36697976

[12] Yang L, Li Z, Xu Z, Zhang B, Liu A, He Q, et al. Protective effects of cannabinoid type 2 receptor activation against microglia overactivation and neuronal pyroptosis in sepsis-associated encephalopathy. Neuroscience. 2022;493:99–108. 10.1016/j.neuroscience.2022.04.011. 35460837 10.1016/j.neuroscience.2022.04.011

[13] Yu TS, Cheng ZH, Li LQ, Zhao R, Fan YY, Du Y, et al. The cannabinoid receptor type 2 is time-dependently expressed during skeletal muscle wound healing in rats. Int J Leg Med. 2010;124:397–404. 10.1007/s00414-010-0465-1. 10.1007/s00414-010-0465-1 20535492

[14] Jiang P, Wang L, Zhang M, Zhang M, Wang C, Zhao R, et al. Cannabinoid type 2 receptor manipulates skeletal muscle regeneration partly by regulating macrophage M1/M2 polarization in IR injury in mice. Life Sci. 2020;256:117989. 10.1016/j.lfs.2020.117989. 32565250 10.1016/j.lfs.2020.117989

[15] Li S, Sun Y, Song M, Song Y, Fang Y, Zhang Q et al. NLRP3/caspase-1/GSDMD-mediated pyroptosis exerts a crucial role in astrocyte pathological injury in mouse model of depression. JCI Insight. 2021;6. 10.1172/jci.insight.146852. 10.1172/jci.insight.146852 PMC8675200 34877938

[16] Coll RC, Schroder K, Pelegrin P. NLRP3 and pyroptosis blockers for treating inflammatory diseases. Trends Pharm Sci. 2022;43:653–68. 10.1016/j.tips.2022.04.003. 35513901 10.1016/j.tips.2022.04.003

[17] Liu X, Zhang Z, Ruan J, Pan Y, Magupalli VG, Wu H, et al. Inflammasome-activated gasdermin D causes pyroptosis by forming membrane pores. Nature. 2016;535:153–8. 10.1038/nature18629. 27383986 10.1038/nature18629 PMC5539988

[18] Aki T, Funakoshi T, Unuma K, Uemura K. Inverse regulation of GSDMD and GSDME gene expression during LPS-induced pyroptosis in RAW264.7 macrophage cells. Apoptosis. 2022;27:14–21. 10.1007/s10495-022-01708-1. 35006493 10.1007/s10495-022-01708-1

[19] Dubyak GR, Miller BA, Pearlman E. Pyroptosis in neutrophils: multimodal integration of inflammasome and regulated cell death signaling pathways. Immunol Rev. 2023;314:229–49. 10.1111/imr.13186. 36656082 10.1111/imr.13186 PMC10407921

[20] Yan J, Zhang J, Wang Y, Liu H, Sun X, Li A, et al. Rapidly inhibiting the inflammatory cytokine storms and restoring cellular homeostasis to alleviate sepsis by blocking pyroptosis and mitochondrial apoptosis pathways. Adv Sci. 2023;10:e2207448. 10.1002/advs.202207448. 10.1002/advs.202207448 PMC10190643 36932048

[21] Yan B, Zhang Y, Liang C, Liu B, Ding F, Wang Y, et al. Stem cell-derived exosomes prevent pyroptosis and repair ischemic muscle injury through a novel exosome/circHIPK3/ FOXO3a pathway. Theranostics. 2020;10:6728–42. 10.7150/thno.42259. 32550900 10.7150/thno.42259 PMC7295049

[22] Li L, Jiang W, Yu B, Liang H, Mao S, Hu X et al. Quercetin improves cerebral ischemia/reperfusion injury by promoting microglia/macrophages M2 polarization via regulating PI3K/Akt/NF-κB signaling pathway. Biomed Pharmacotherapy. 2023;168. 10.1016/j.biopha.2023.115653. 10.1016/j.biopha.2023.115653 37812891

[23] Liu JB, Jia SS, Yang Y, Piao LH, Wang ZY, Jin ZZ et al. Exercise induced meteorin-like protects chondrocytes against inflammation and pyroptosis in osteoarthritis by inhibiting PI3K/Akt/NF-κB and NLRP3/caspase-1/GSDMD signaling. Biomed Pharmacotherapy. 2023;158. 10.1016/j.biopha.2022.114118. 10.1016/j.biopha.2022.114118 36527845

[24] Song CC, Yang ZX, Dong D, Xu JW, Wang J, Li H, et al. miR-483 inhibits bovine myoblast cell proliferation and differentiation via signal pathway. J Cell Physiol. 2019;234:9839–48. 10.1002/jcp.27672. 30422322 10.1002/jcp.27672

[25] Li J, Dong S, Quan S, Ding S, Zhou X, Yu Y et al. Nuciferine reduces inflammation induced by cerebral ischemia-reperfusion injury through the PI3K/Akt/NF-κB pathway. Phytomedicine. 2024:125. 10.1016/j.phymed.2023.155312. 10.1016/j.phymed.2023.155312 38232541

[26] Guldenpfennig C, Teixeiro E, Daniels M. NF-kB’s contribution to B cell fate decisions. Front Immunol. 2023;14:1214095. 10.3389/fimmu.2023.1214095. 37533858 10.3389/fimmu.2023.1214095 PMC10391175

[27] Robinson N, Ganesan R, Hegedűs C, Kovács K, Kufer TA, Virág L. Programmed necrotic cell death of macrophages: Focus on pyroptosis, necroptosis, and parthanatos. Redox Biol. 2019;26:101239. 10.1016/j.redox.2019.101239. 31212216 10.1016/j.redox.2019.101239 PMC6582207

[28] Eming SA, Wynn TA, Martin P. Inflammation and metabolism in tissue repair and regeneration. Science. 2017;356:1026–30. 10.1126/science.aam7928. 28596335 10.1126/science.aam7928

[29] Pang BPS, Iu ECY, Hang MJ, Chan WS, Tse MCL, Yeung CTY et al. Deficiency of muscle-generated brain-derived neurotrophic factor causes inflammatory myopathy through reactive oxygen species-mediated necroptosis and pyroptosis. Redox Biol. 2024;78. 10.1016/j.redox.2024.103418. 10.1016/j.redox.2024.103418 PMC11602578 39531828

[30] Chi ZX, Chen S, Yang DH, Cui WY, Lu Y, Wang Z, et al. Gasdermin D-mediated metabolic crosstalk promotes tissue repair. Nature. 2024;634:1168–77. 10.1038/s41586-024-08022-7. 39260418 10.1038/s41586-024-08022-7

[31] You Z, Huang X, Xiang Y, Dai J, Xu L, Jiang J, et al. Ablation of NLRP3 inflammasome attenuates muscle atrophy via inhibiting pyroptosis, proteolysis and apoptosis following denervation. Theranostics. 2023;13:374–90. 10.7150/thno.74831. 36593964 10.7150/thno.74831 PMC9800723

[32] Zhang L, Wang X, Yu W, Ying J, Fang P, Zheng Q, et al. CB2R activation regulates TFEB-mediated autophagy and affects lipid metabolism and inflammation of astrocytes in POCD. Front Immunol. 2022;13:836494. 10.3389/fimmu.2022.836494. 35392078 10.3389/fimmu.2022.836494 PMC8981088

[33] Gaul S, Leszczynska A, Alegre F, Kaufmann B, Johnson CD, Adams LA, et al. Hepatocyte pyroptosis and release of inflammasome particles induce stellate cell activation and liver fibrosis. J Hepatol. 2021;74:156–67. 10.1016/j.jhep.2020.07.041. 32763266 10.1016/j.jhep.2020.07.041 PMC7749849

[34] Amenta PS, Jallo JI, Tuma RF, Hooper DC, Elliott MB. Cannabinoid receptor type-2 stimulation, blockade, and deletion alter the vascular inflammatory responses to traumatic brain injury. J Neuroinflamm. 2014;11: 10.1186/s12974-014-0191-6. 10.1186/s12974-014-0191-6 PMC4248435 25416141

[35] Luo M, Zhao FK, Cheng H, Su M, Wang YM. Macrophage polarization: an important role in inflammatory diseases. Front Immunol. 2024;15. 10.3389/fimmu.2024.1352946. 10.3389/fimmu.2024.1352946 PMC11039887 38660308

[36] Yang S, Yin Y, Sun Y, Ai D, Xia X, Xu X, et al. AZGP1 aggravates macrophage M1 polarization and pyroptosis in periodontitis. J Dent Res. 2024;103:631–41. 10.1177/00220345241235616. 38491721 10.1177/00220345241235616

[37] Xiong YL, Zhang ZH, Liu SY, Shen LS, Zheng LH, Ding LG et al. Lupeol alleviates autoimmune myocarditis by suppressing macrophage pyroptosis and polarization via PPARα/LACC1/NF-κB signaling pathway. Phytomedicine. 2024;123. 10.1016/j.phymed.2023.155193. 10.1016/j.phymed.2023.155193 37976692

[38] Pryce BR, Oles A, Talbert EE, Romeo MJ, Vaena S, Sharma S, et al. Muscle inflammation is regulated by NF-κB from multiple cells to control distinct states of wasting in cancer cachexia. Cell Rep. 2024;43:114925. 10.1016/j.celrep.2024.114925. 39475511 10.1016/j.celrep.2024.114925 PMC11774514

[39] Hashiesh HM, Azimullah S, Nagoor Meeran MF, Saraswathiamma D, Arunachalam S, Jha NK, et al. Cannabinoid 2 receptor activation protects against diabetic cardiomyopathy through inhibition of AGE/RAGE-induced oxidative stress, fibrosis, and inflammasome activation. J Pharm Exp Ther. 2024;391:241–57. 10.1124/jpet.123.002037. 10.1124/jpet.123.002037 38955492

[40] Zhang M, Guo B, Zhang X, Han D, Lv L, Yan X, et al. IFP35, a novel DAMP, aggravates neuroinflammation following acute ischemic stroke via TLR4/NF-κB/NLRP3 signaling. J Neuroinflammation. 2025;22:164. 10.1186/s12974-025-03492-6. 40563086 10.1186/s12974-025-03492-6 PMC12188676

[41] Qin H, Luo Z, Sun Y, He Z, Qi B, Chen Y, et al. Low-intensity pulsed ultrasound promotes skeletal muscle regeneration via modulating the inflammatory immune microenvironment. Int J Biol Sci. 2023;19:1123–45. 10.7150/ijbs.79685. 36923940 10.7150/ijbs.79685 PMC10008697

[42] Tidball JG. Regulation of muscle growth and regeneration by the immune system. Nat Rev Immunol. 2017;17:165–78. 10.1038/nri.2016.150. 28163303 10.1038/nri.2016.150 PMC5452982

[43] Dubuisson N, Versele R, de Carrizosa M, Selvais CM, Brichard SM, Abou-Samra M. Walking down skeletal muscle lane: from inflammasome to disease. Cells. 2021;10. 10.3390/cells10113023. 10.3390/cells10113023 PMC8616386 34831246

[44] Zhang M, Zhang M, Wang L, Yu T, Jiang S, Jiang P, et al. Activation of cannabinoid type 2 receptor protects skeletal muscle from ischemia-reperfusion injury partly via Nrf2 signaling. Life Sci. 2019;230:55–67. 10.1016/j.lfs.2019.05.056. 31128135 10.1016/j.lfs.2019.05.056

[45] Tian ZL, Wang RL, Yang QF, Qin ZQ, Dong HW, Zou DH, et al. Detection of multiple biomarkers associated with satellite cell fate in the contused skeletal muscle of rats for wound age estimation. Int J Leg Med. 2023;137:875–86. 10.1007/s00414-023-02971-w. 10.1007/s00414-023-02971-w 36797435

[46] Steib CJ, Gmelin L, Pfeiler S, Schewe J, Brand S, Goke B, et al. Functional relevance of the cannabinoid receptor 2 - heme oxygenase pathway: a novel target for the attenuation of portal hypertension. Life Sci. 2013;93:543–51. 10.1016/j.lfs.2013.08.018. 24007798 10.1016/j.lfs.2013.08.018

[47] Li J, Hao J-H, Yao D, Li R, Li X-F, Yu Z-Y, et al. Caspase-1 inhibition prevents neuronal death by targeting the canonical inflammasome pathway of pyroptosis in a murine model of cerebral ischemia. CNS Neurosci Therapeutics. 2020;26:925–39. 10.1111/cns.13384. 10.1111/cns.13384 PMC7415206 32343048

[48] Lin Y, Zhu X, Li Y, Dou Y, Wang J, Qi R, et al. LY294002 ameliorates psoriatic skin inflammation in mice via blocking the Notch1/Hes1-PTEN/AKT/IL-17A feedback loop. Clin Exp Immunol. 2023;213:114–24. 10.1093/cei/uxad025. 36840628 10.1093/cei/uxad025 PMC10324552

[49] Liu X, Ren X, Zhou L, Liu K, Deng L, Qing Q, et al. Tollip orchestrates macrophage polarization to alleviate intestinal mucosal inflammation. J Crohns Colitis. 2022;16:1151–67. 10.1093/ecco-jcc/jjac019. 35134154 10.1093/ecco-jcc/jjac019

[50] Tonkin J, Temmerman L, Sampson RD, Gallego-Colon E, Barberi L, Bilbao D, et al. Monocyte/macrophage-derived IGF-1 orchestrates murine skeletal muscle regeneration and modulates autocrine polarization. Mol Ther. 2015;23:1189–1200. 10.1038/mt.2015.66. 25896247 10.1038/mt.2015.66 PMC4817788

