# Supplementary material for: Cannabinoid type 2 receptor regulates skeletal muscle regeneration by NLRP3-GSDMD mediated macrophage pyroptosis after injury
Source: Cell Death Discov. 2026 Mar 27;12:198. doi: 10.1038/s41420-026-03077-z (PMC13144689; doi:10.1038/s41420-026-03077-z)
Supplement: Supplementary file 1 — SUPPLEMENTAL MATERIAL [file 41420_2026_3077_MOESM1_ESM.docx]

**Supplementary Figure.1**

**
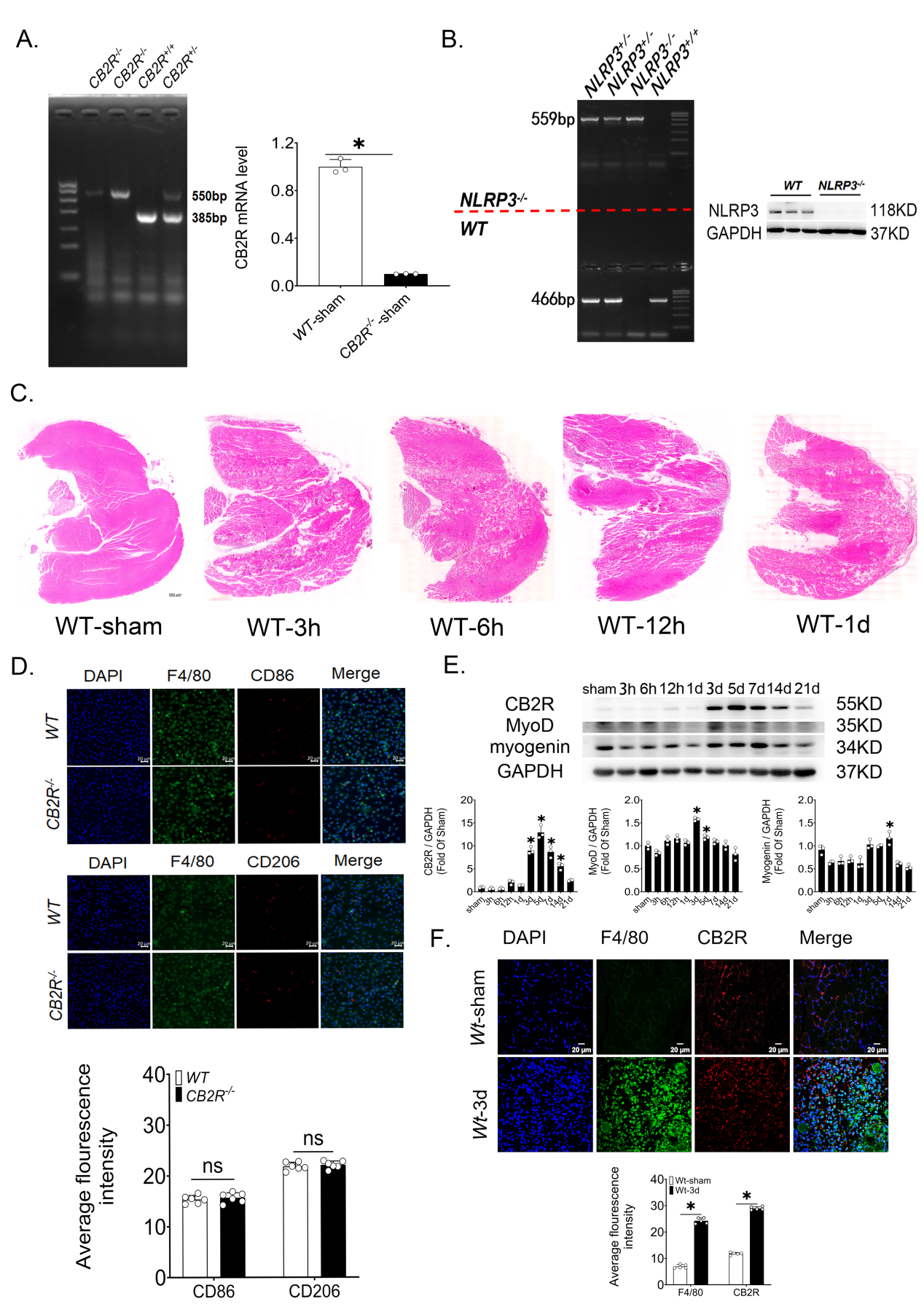
**

**Supplementary Figure.1 Mouse genotype verification, contusion model verification and CB2R knockout aggravate the inflammatory response after skeletal muscle injury**

(A) Representative bands of agarose gel electrophoresis of *CB2R^-/-^* mouse genotype and qPCR results of CB2R. (B) *NLRP3^-/-^* mouse genotype verification. (C) Verification of stability, uniformity and success of skeletal muscle injury model, scale bar=200μm. (D) Polarization of primitive peritoneal macrophages, scale bar=20μm, n=5, ns, no significance. (E) Temporal expression of CB2R, MyoD and myogenin proteins, n=3, data are represented as mean±SD, **p*<0.05 vs. sham group. (F) The changes in CB2R expression in macrophages after skeletal muscle injury, n=5, data are represented as mean±SD, **p*<0.05 *Wt*-sham vs. *Wt*-3d group.

**Supplementary Figure.2**

**
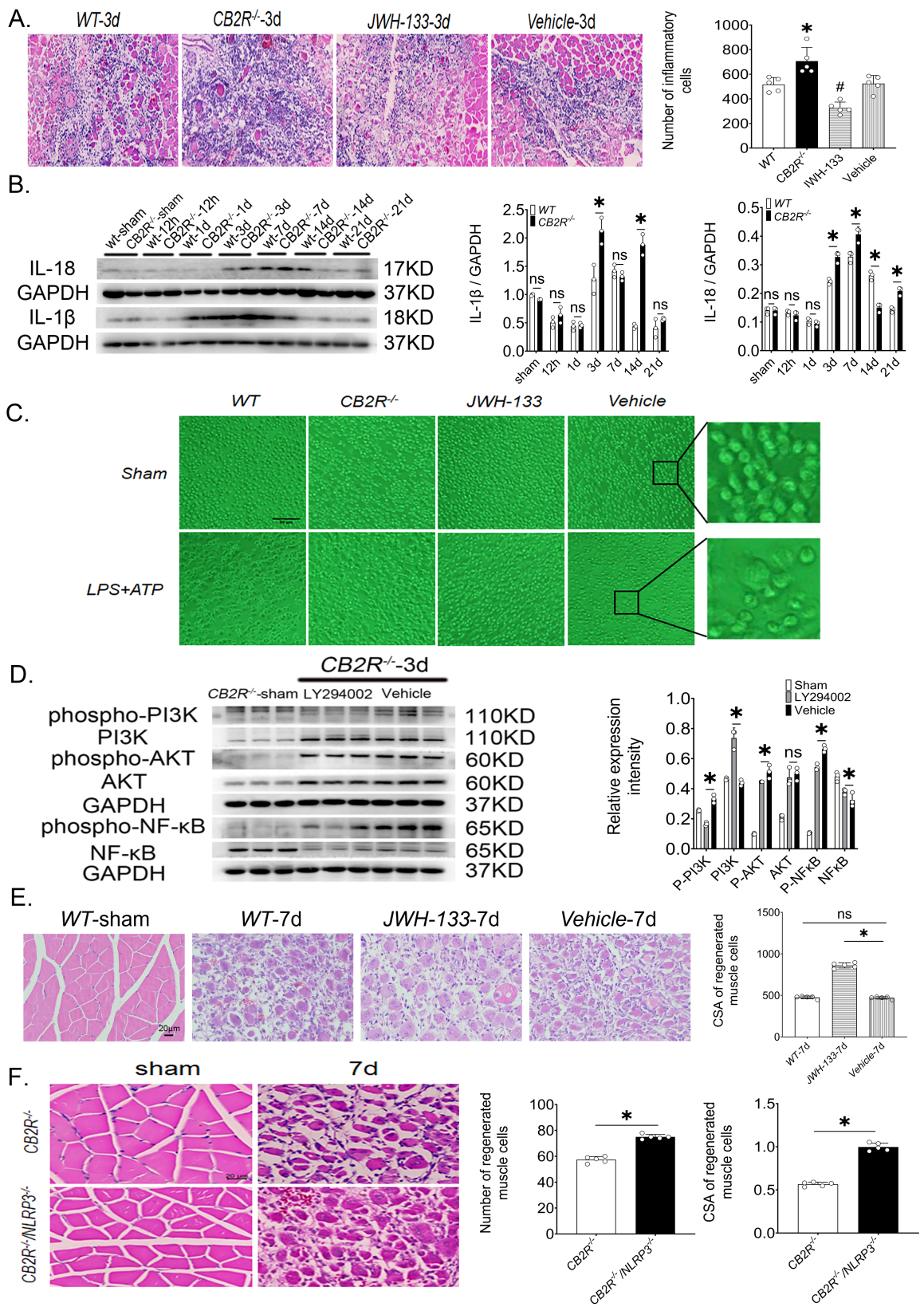
**

**Supplementary Figure.2 Temporal expression pattern of CB2R and optical microscopy pictures of peritoneal macrophages.**

(A) Infiltration of inflammatory cells in the injury area, scale bar=100μm, n=5, data are represented as mean±SD, **p*<0.05 vs. sham group, #*p*<0.05 vs. Vehicle group. (B) Temporal expression of IL-18 and IL-1β after skeletal muscle injury in WT mice and *CB2R^-/-^* mice, n=3, data are represented as mean±SD, **p*<0.05 vs. *CB2R^-/-^* group. ns, no significance. (C) Representative light microscope pictures of peritoneal macrophages, scale bar=20μm. (D) Protein expression levels of PI3K, phospho-PI3K, AKT, phospho-AKT, NF-κB and phospho-NF-κB. n=3, **p*<0.05 vs. Vehicle group. (E) H&E representative pictures of skeletal muscle regeneration, scale bar=20μm. n=5, **p*<0.05 *Vehicle-*7d vs. *JWH-133*-7d group. (F) H&E representative pictures of skeletal muscle regeneration, scale bar=20μm, n=5, data are represented as mean±SD, **p*<0.05 *CB2R^-/-^* vs. *CB2R^-/-^/NLRP3^-/-^* group.

**Supplementary Figure.3**

**
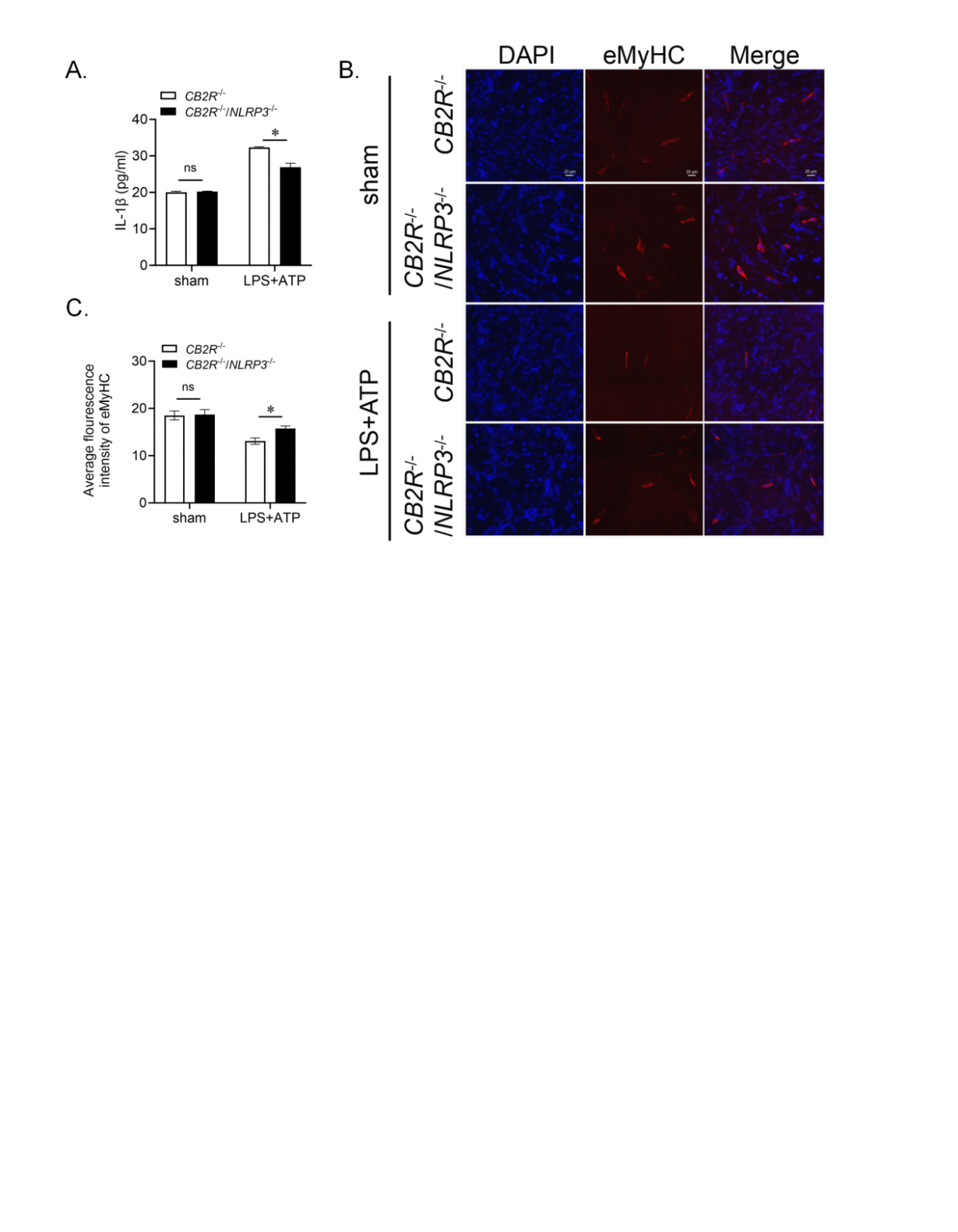
**

**Supplementary Figure.3 CB2R regulates C2C12 myogenic differentiation in an NLRP3-dependent manner.**

(A) The release of IL-1β from macrophages after LPS+ATP treatment, n=4, **p*<0.05 *Cb2r^-/-^/Nlrp3^-/-^* vs. *Cb2r^-/-^* group. (B, C) Expression of eMyHC in C2C12 after treatment with macrophage-conditioned medium, scale bar=20μm, n=3, **p*<0.05 sham vs. LPS+ATP group. **p*<0.05 *Cb2r^-/-^/Nlrp3^-/-^* vs. *Cb2r^-/-^* group

**Supplementary Figure.4**

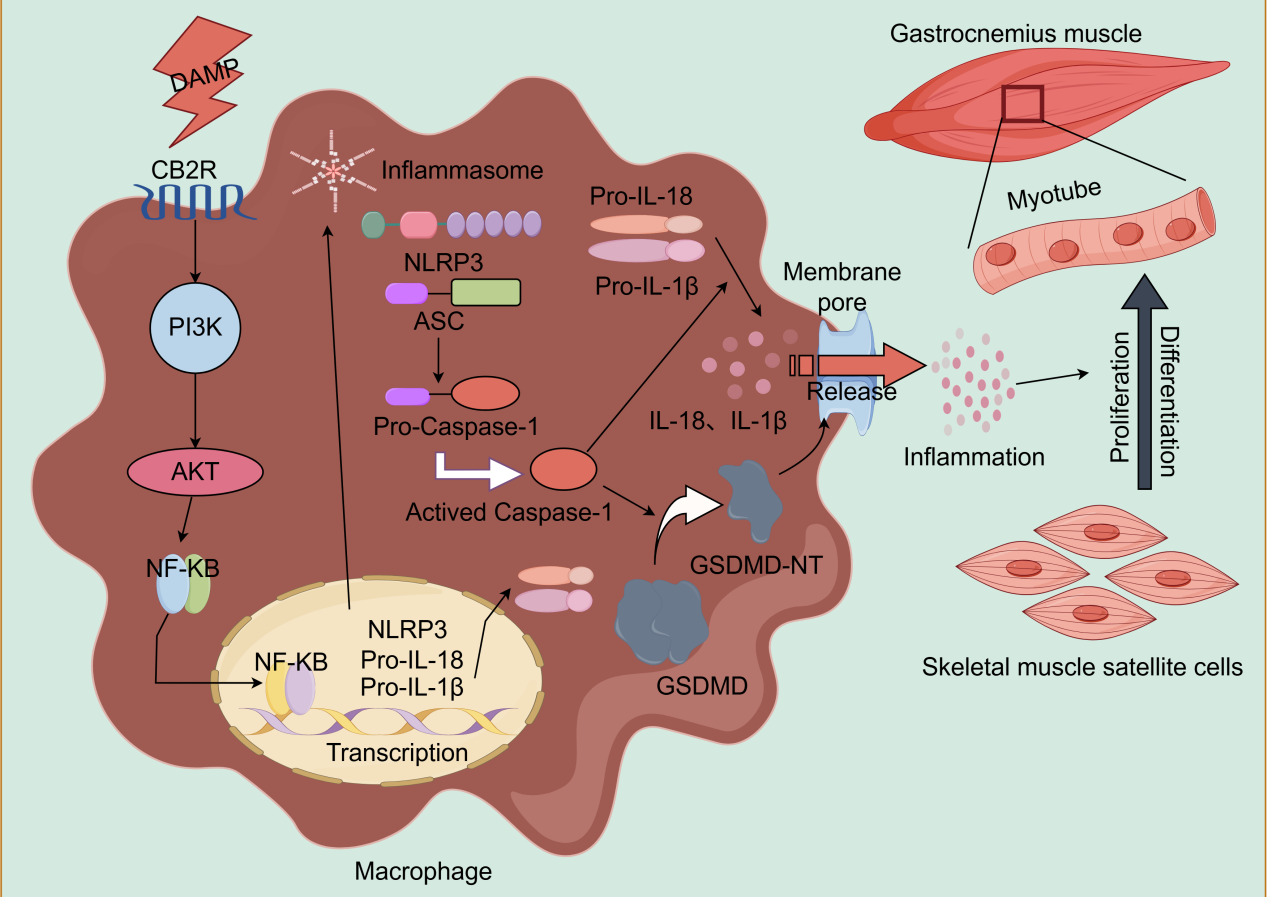


**Supplementary Figure.4 Schematic drawing of the major finding**

This study demonstrates that CB2R facilitates NLRP3-GSDMD mediated macrophage pyroptosis via the PI3K/AKT/NF-κB pathway, leading to increased release of IL-1β following skeletal muscle injury, aggravate the inflammatory response, and thereby inhibit regeneration after injury.

**Supplementary Table.1**

| GENE NAME | FORWORD | REVERSE |
| --- | --- | --- |
| *NLRP3* | CTCCAACCATTCTCTGACCAG | ACAGATTGAAGTAAGGCCGG |
| *ASC* | AGCAAGAGTAAAAGGTGACCG | TTGAGTTCATCCCCTGACAAG |
| *caspase-1* | TCTGTATTCACGCCCTGTTG | GATAAATTGCTTCCTCTTTGCCC |
| *GSDMD* | CTATGCCTCCCTGTTCCTATTG | TGAGTATGGTTCTTGGCTTCC |
| *IL-1β* | ACGGACCCCAAAAGATGAAG | TTCTCCACAGCCACAATGAG |
| *GAPDH* | CTTTGTCAAGCTCATTTCCTGG | TCTTGCTCAGTGTCCTTGC |
