# Supplementary material for: Cannabinoid type 2 receptor regulates skeletal muscle regeneration by NLRP3-GSDMD mediated macrophage pyroptosis after injury
Source: Cell Death Discov. 2026 Mar 27;12:198. doi: 10.1038/s41420-026-03077-z (PMC13144689; doi:10.1038/s41420-026-03077-z)

Figure.1.d

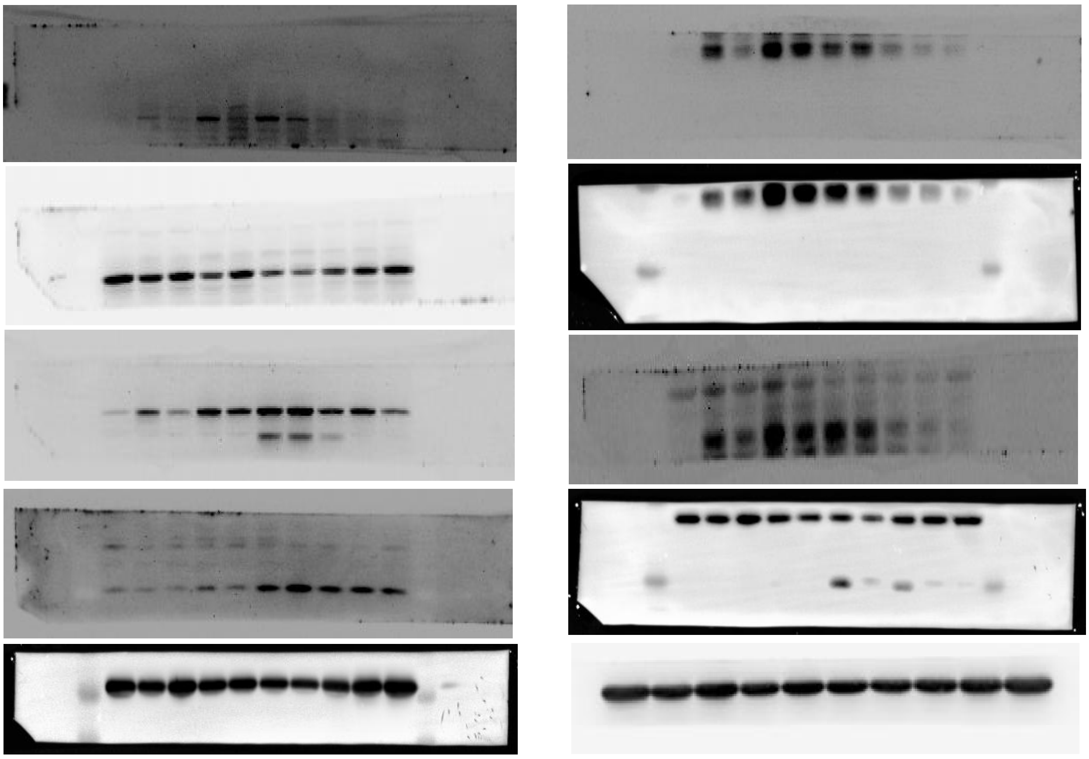

Figure.2.b

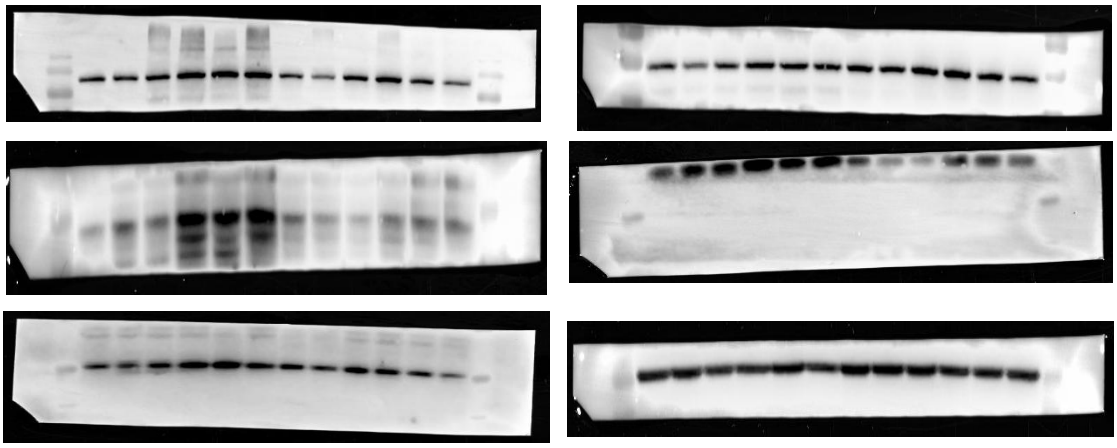

Figure.2.e

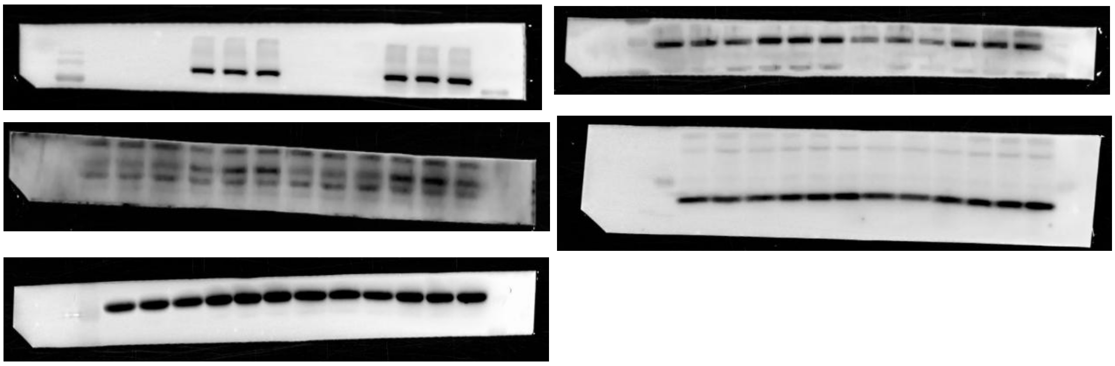

Figure.3.a

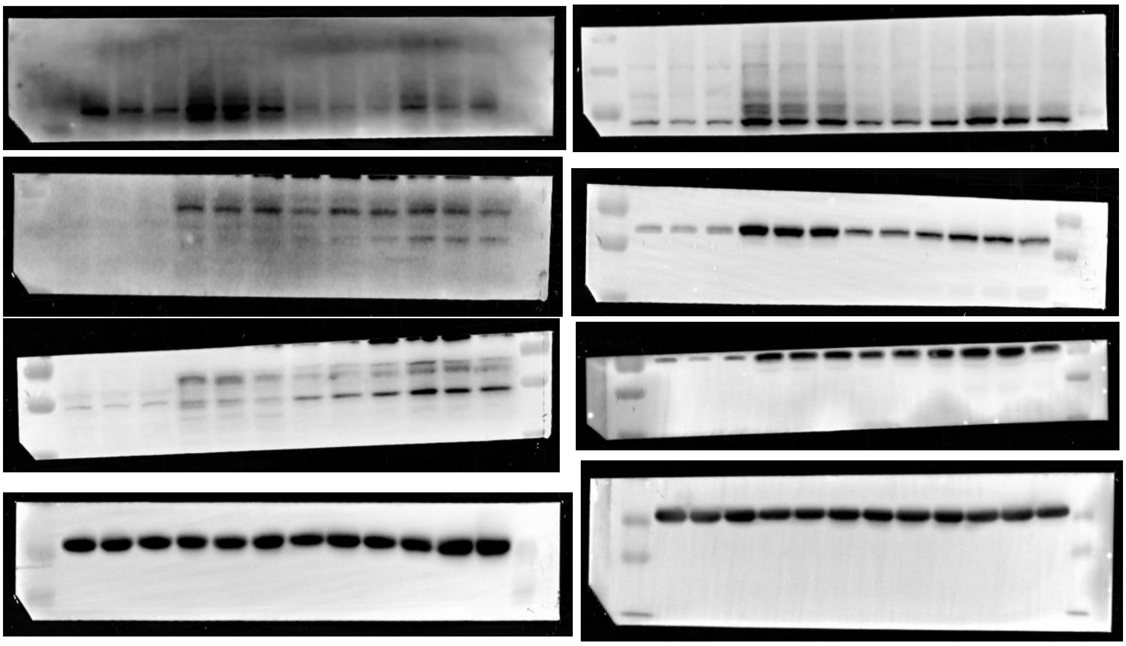

Figure.3.e

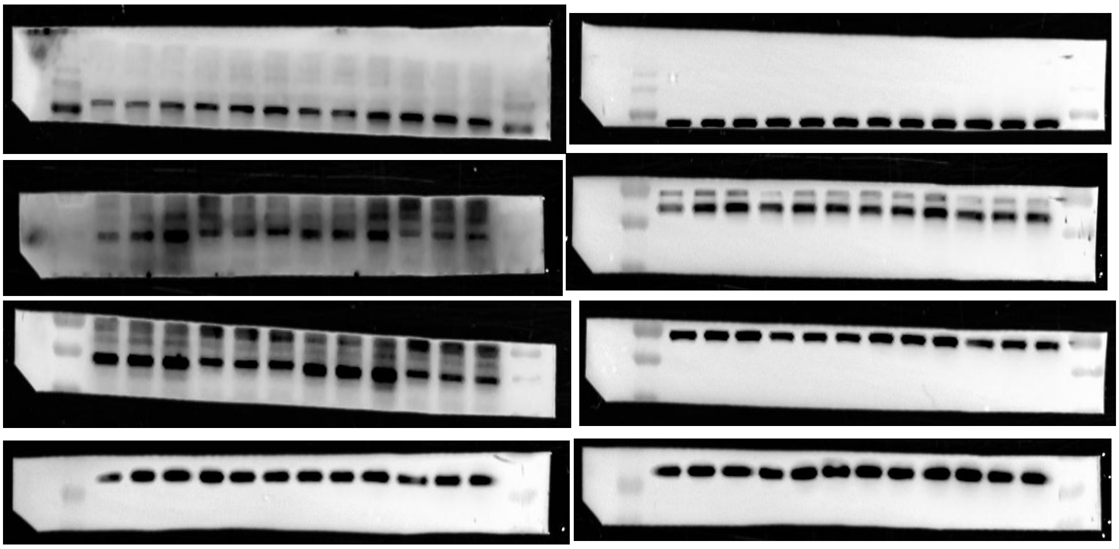

Figure.3.f

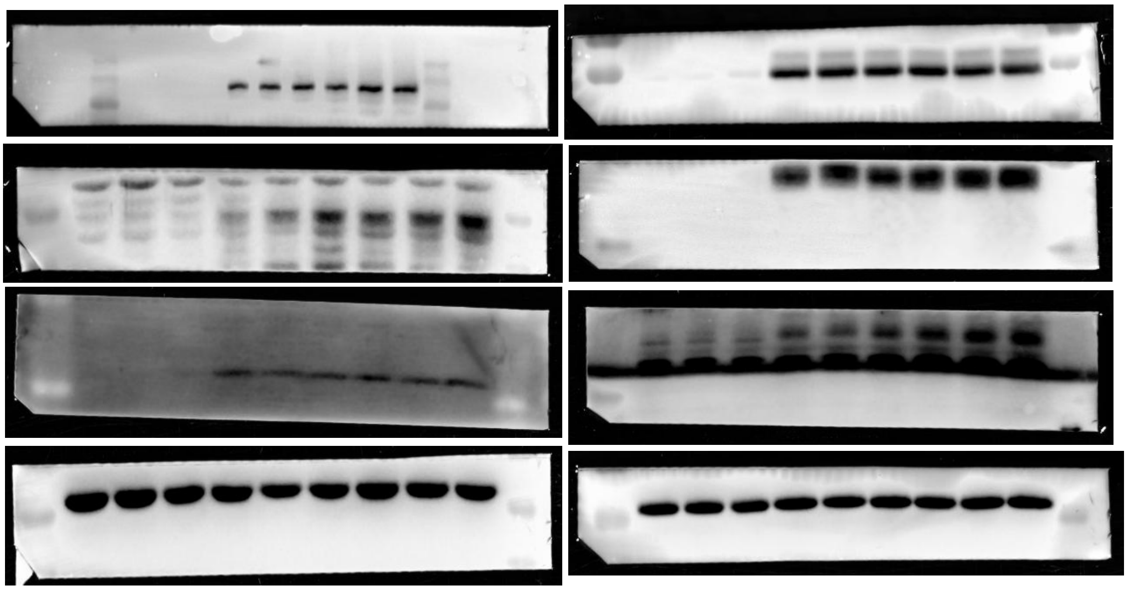

Figure.4.b

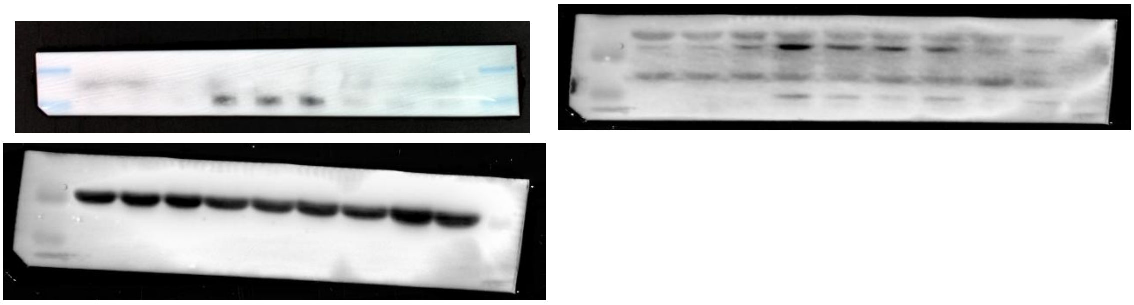

Figure.4.e

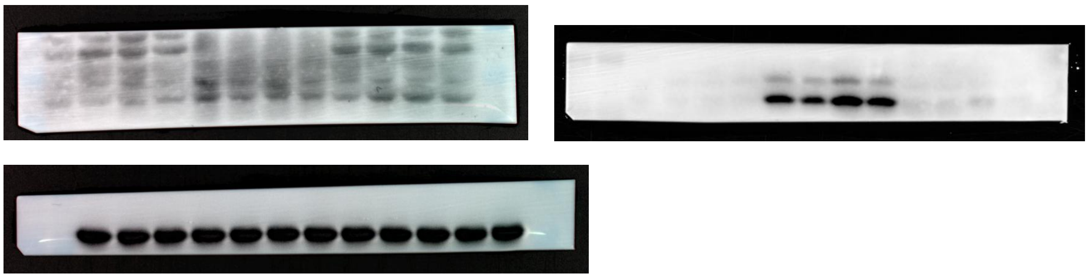

Figure.5.e

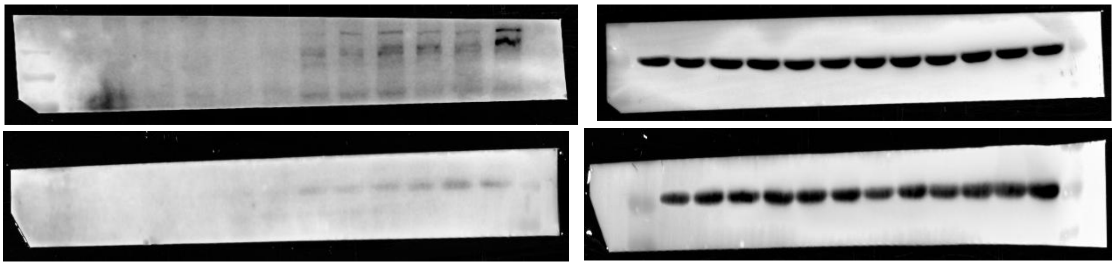

Figure.6.b

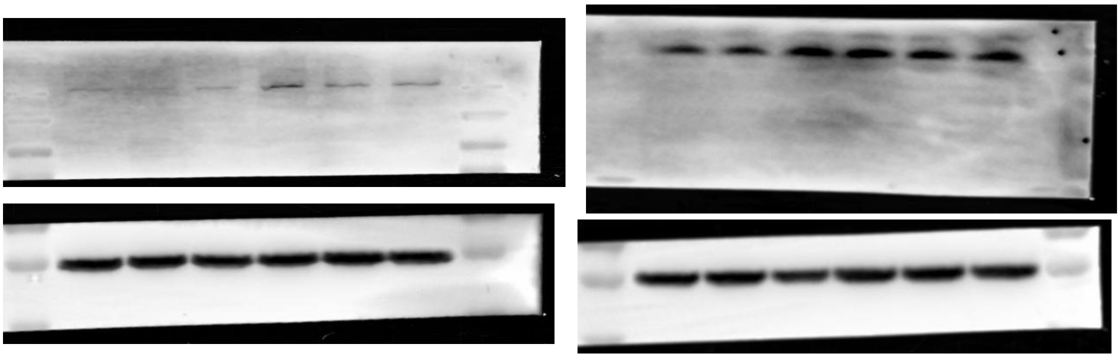

Figure.6.e

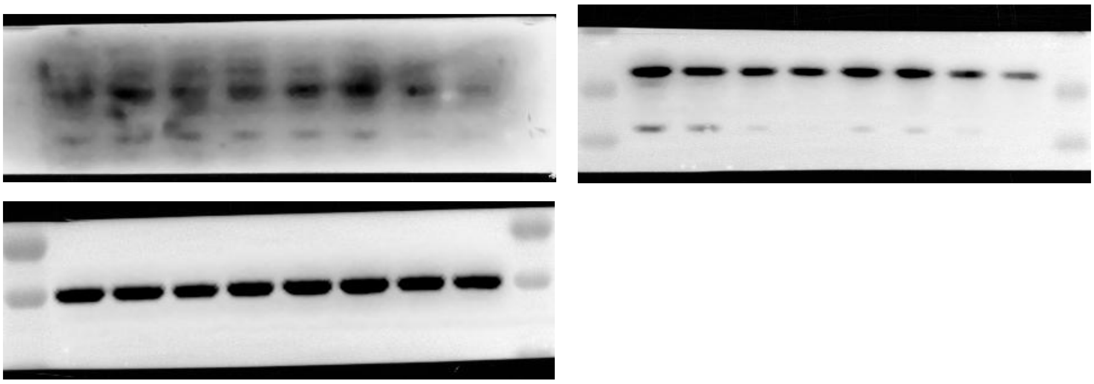

Supplement Figure.1F

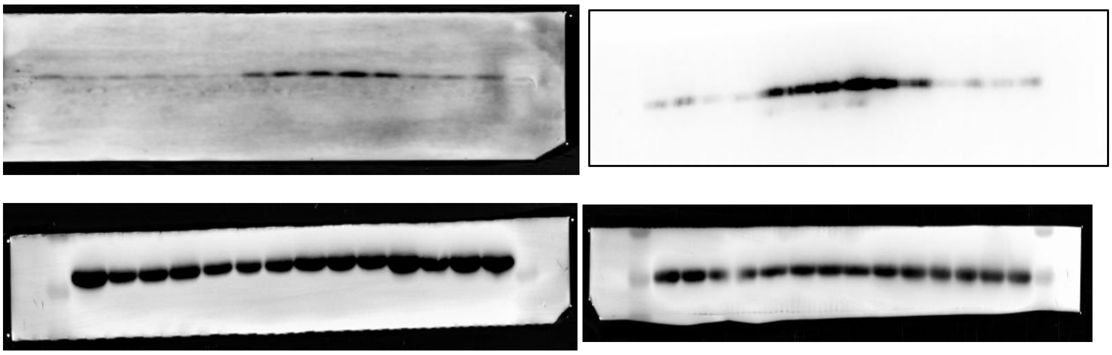

Supplement Figure.2A

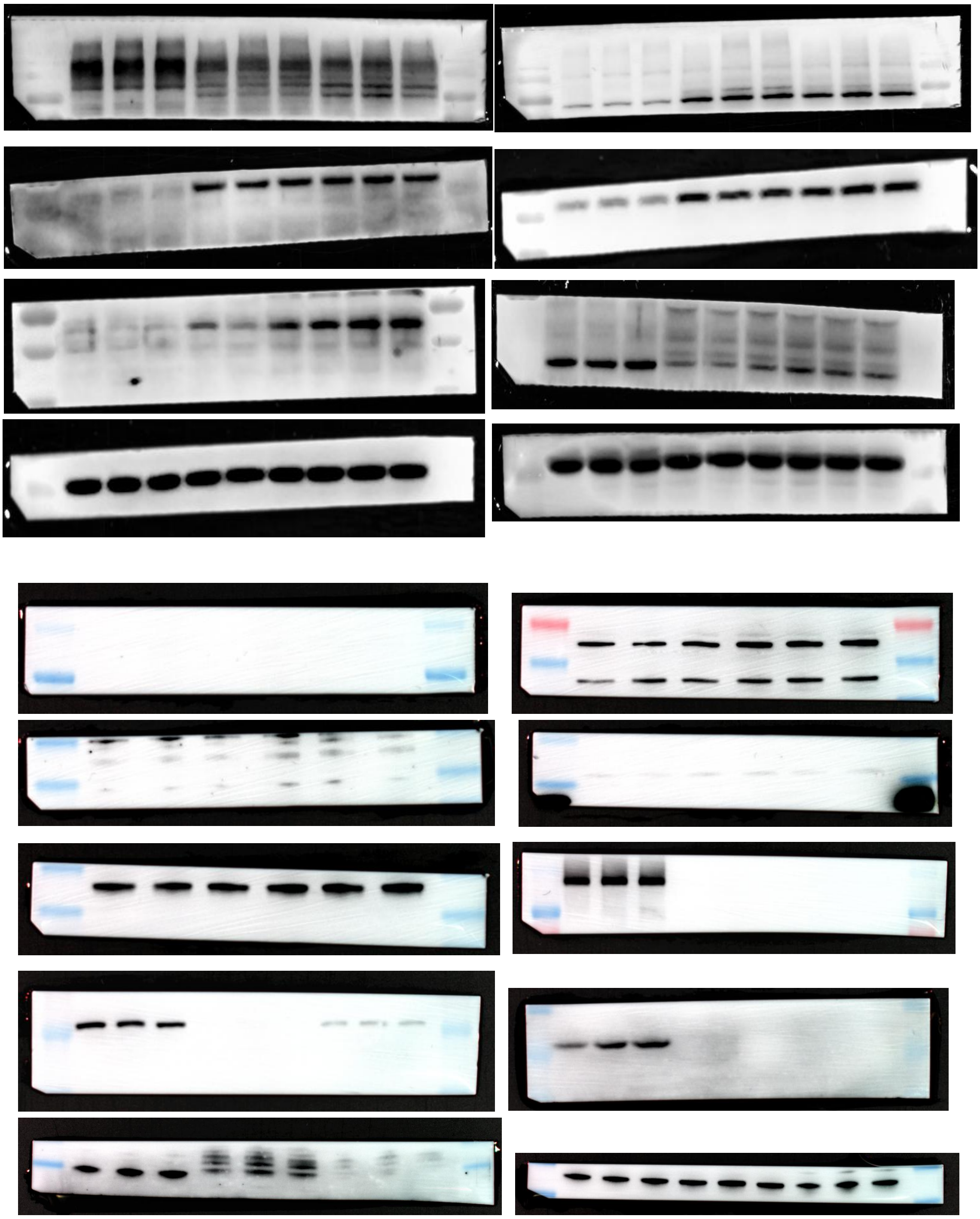

Supplement Figure.2B

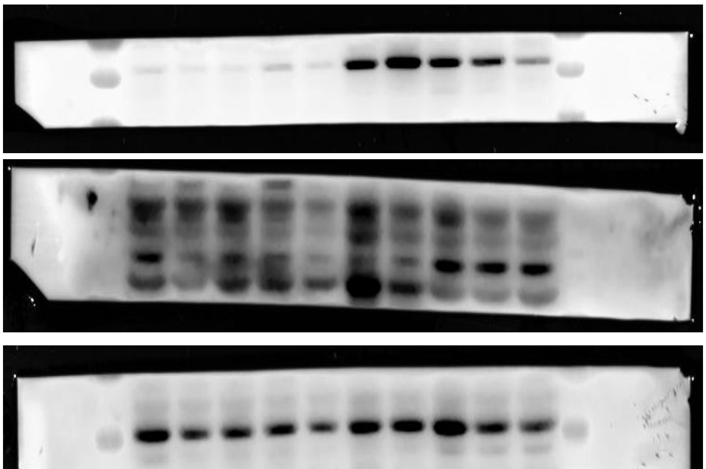

Supplement: Supplementary file 2 — Original western blots [file 41420_2026_3077_MOESM2_ESM.pdf]
